# Acceleration of the Relativistic Dirac–Kohn–Sham Method with GPU: A Pre-Exascale Implementation of BERTHA and PyBERTHA

**DOI:** 10.1021/acs.jctc.4c01759

**Published:** 2025-03-21

**Authors:** Loriano Storchi, Laura Bellentani, Jeff Hammond, Sergio Orlandini, Leonardo Pacifici, Nicoló Antonini, Leonardo Belpassi

**Affiliations:** † Dipartimento di Farmacia, Università G. d’Annunzio Chieti-Pescara, via dei Vestini 31, 66100 Chieti, Italy; ‡ CNR Institute of Chemical Science and Technologies “Giulio Natta” (CNR-SCITEC), via Elce di Sotto 8, 06123 Perugia, Italy; § CINECA, Via dei Tizi 6/b, 00185 Roma, RM, Italy; ∥ NVIDIA Helsinki Oy, 00180 Helsinki, Finland; ⊥ Dipartimento di Chimica, Università degli Studi di Perugia, via Elece di Sotto 8, 06123 Perugia, Italy

## Abstract

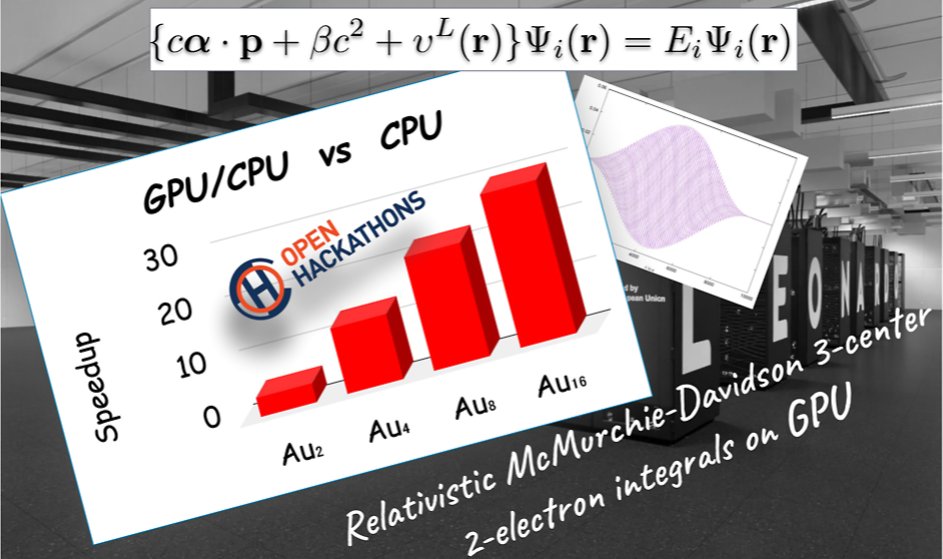

In this paper, we present the recent advances in the computation of the Dirac–Kohn–Sham (DKS) method of the BERTHA code. We show here that the simple underlined structure of the FORTRAN code also favors efficient porting of the code to GPUs, leading to a particularly efficient hybrid CPU/GPU implementation (OpenMP/OpenACC), where the most computationally intensive part for DKS matrix evaluation (three-center two-electron integrals evaluated via the McMurchie–Davidson scheme) is efficiently offloaded to the GPU via compiler directives based on the OpenACC programming model. This scheme in combination with the use of a linear algebra library optimized for GPUs (cuBLAS, cuSOLVER) significantly accelerates the DKS calculations. In addition, the low-level integral kernel developed here at FORTRAN level was used to port our real-time DKS (RT-TDDKS) implementation based on Python (PyBERTHART) for the utilization of the GPU. The results obtained on the new Tier-0 EuroHPC supercomputer (LEONARDO) of the CINECA Supercomputing Centre with a single NVIDIA A100 card are very satisfactory. We achieve a speedup up to 30 for Au_16_ in a single-point DKS energy calculation and up to 10 for the Au_8_ systems in an RT-TDDKS calculation, compared to our OpenMP (i.e., CPU only) parallel implementation (with 32 cores). The approach presented here is very general and, to our knowledge, represents the first port of a Python API to GPUs based on a FORTRAN kernel for the evaluation of two-electron integrals. The implementation is currently limited to the use of a single GPU accelerator, but future paths to an actual exascale implementation are discussed.

## 1 Introduction

Today is an exciting time for computational science, as computing power is increasing and HPC (High Performance Computing) systems have even reached the exascale^1^ class (10^18^ floating point operations per second). HPC systems with extremely high computing power are therefore an important factor for major technological progress and innovation and a strategic resource for countries. The first three exascale HPC systems on the TOP500 list are located in the USA (*El Capitan*, *Frontier* and *Aurora* HPC systems installed at Lawrence Livermore National Laboratory in California, Oak Ridge National Laboratory and Argonne National Laboratory respectively) and are already in production. However, the lack of Chinese HPC systems affects the actual completeness of the Top500 list. At the end of this year, the DIGITAL Europe program will also invest in the commissioning of JUPITER, the first European exascale supercomputer.

Developments in HPC and computational science are having a significant impact on all scientific fields that utilize computational resources to solve complex problems, including molecular and material sciences. The leading data centers (and now desktop workstations) have deployed hardware consisting of heterogeneous compute nodes with multicore CPUs and accelerators, with much of the acceleration provided by the graphics processing units (GPUs). GPUs are designed to run fine-grained threads that are short-lived and do minimal work at a time. In the general GPU programming model, fine-grained computational workloads must be organized into thread blocks to offload them to the GPU. To achieve high performance, a problem must be defined in terms of a large number of similar tasks in order to effectively utilize the SIMT-based GPU architecture (Single Instruction Multiple Threads). It is therefore not surprising that considerable efforts have been made to efficiently adapt conventional quantum chemistry algorithms or to develop them from scratch in order to port them to GPUs.

In 2008, two groundbreaking papers by Yasuda^2^ and Ufimtsev and Martinez^3^ showed for the first time that GPUs (then still with single-precision arithmetic) could be useful in quantum chemistry by accelerating the calculations of two-electron repulsion integrals on Gaussian functions with fixed small angular momentum. In 2010, the first double precision GPU was available (NVIDIA, Fermi) and since then there has been an impressive hardware development of GPUs for HPC computing (also fuelled by the recent interest in AI and generative AI). In addition to NVIDIA, AMD and Intel have also become GPU developers.

The real benefits of using GPUs in quantum chemistry are now well documented^4^ and many codes for molecular^5−11^ and materials science^12−14^ have been or are expected to be accelerated by porting to GPUs. An amazing example of the maturity and potential of this field is the recent massively parallel GPU implementation of the nuclear gradient of the TDDFT method, which uses Gaussian atomic basis sets and allows the study of electronic excited states of small protein (with thousands of atoms).^15^

There are currently various strategies for porting code to GPUs. It is up to the developers and the specificity of the code to choose a programming model that balances the requirements of portability, performance and maintainability for future developments. Low-level programming models based on CUDA or OpenCL, for example, have a high parallel programming capability with explicit control and management of how the computational problem needs to be distributed across the hardware. If the implementation is well designed, it can achieve the highest performance, although the low-level nature of these approaches can make codes less portable and more complicated to maintain.

A suitable alternative nowadays are high-level programming models based on compiler directives (in the form of pragmas in C/C++ or comments in Fortran), which provide additional information about the creation and transfer of data from the host to the device and the optimization. Compilers support directives (e.g., OpenACC and OpenMP) to offload calculations and manage data transfer, and now have effective debugging tools. In many cases, a high-level programming model is expected to be more portable across architectures and avoid duplication of source code. For linear algebra, there are efficient and well-supported libraries for GPUs (cuBLAS, cuSOLVER library for NVIDIA GPUs, rocBLAS, rocSolver for AMD GPUs, or oneMKL library for Intel GPUs) that greatly facilitate portability. In addition, there are a number of newer GPU-accelerated libraries for the evaluation of the electron repulsion integral (ERI) for quantum chemistry that are based on CUDA/C++ (LibintX^16,17^) or use the directives of the compilers (e.g., OpenMP and OpenACC) and also utilize the parallelism of standard languages (with Fortran DO CONCURRENT^18−20^), e.g. the LibERI library.^21^

A relatively simple way to accelerate code developed in a Python-based environment that uses linear algebra is to use libraries such as CuPy,^22^ which rely internally on CUDA routines to offload calculations to devices, but have an interface that is highly compatible and allows a drop-in replacement for Numpy/Scipy functions. CuPy can be used with NVIDIA GPUs and in the latest versions also with ROCm for AMD GPUs. A nice example of development within a Python environment has recently been reported by Kriebel et al.^23^ for the acceleration of Pythonic coupled cluster implementations within the PyBEST code.^24^ Quantum chemistry software working in a Python environment (e.g., PySCF^25^ and Psi4Numpy^26^) are valuable and provide an interactive quantum chemistry framework for reference implementations, rapid prototyping, development and training, while ensuring high efficiency by evaluating the necessary integrals via external integral libraries or interfaces to efficient codes. All Python-level variables can then be manipulated using the NumPy package, providing a suitable framework for development that is clear, readable and easy to test. It also allows integration with other Python-based programmes to create workflows of increasing complexity. The GPU-accelerated PySCF code has only recently been proposed (GPU4PySCF). The porting to GPUs is based on the efficient evaluation of four-center ERIs (based on CUDA kernels) up to *g*-functions,^27^ which have also been extended to 3-center integrals for density fitting using Rys quadrature.^28^

As far as computational costs are concerned, accurate relativistic quantum chemistry methods based on the four-component (4c) Dirac equation pose a particular challenge. Although they are extremely accurate for tracing relativistic effects and include spin–orbit coupling in a variational manner, the full 4c calculations are inherently associated with higher computational costs than analogue nonrelativistic approaches. This is mainly due to the complex matrices that need to be processed, the increased effort required to evaluate the basic variables from the spinor amplitudes and the larger basis sets that are usually required. There are a number of software programmes for relativistic electronic structure that are currently under active development.^29−32^

Despite the computing power offered by GPUs, GPU-accelerated full 4c calculations are still very rare. As notable exceptions, we mention the reimplementation of the core algorithms of relativistic coupled cluster theory^33^ and its frequency-dependent linear response version,^34^ designed for parallel execution on many compute nodes with optional GPU coprocessing performed via the new ExaTENSOR backend.^35^ More recently, Kovtun et al.^36^ off-loads to GPUs the numerical integration of the exchange–correlation potential for exact 2-component density functional theory.

This paper describes our recent efforts to move the most computationally intensive parts of the 4c Dirac–Kohn–Sham (DKS) method implemented in the BERTHA package to the GPU. The BERTHA code is built in Fortran around an efficient algorithm for the analytic evaluation of relativistic electronic repulsion integrals developed by Quiney and Grant^37−39^ which is a relativistic generalization of the well-known McMurchie-Davidson algorithm.^38,40^ The basis functions used are called G-spinors and are two-component spin–orbit coupled functions derived from spherical Gaussian-type functions.^41^ Thanks to its clean original design and an efficient implementation based on variational density fitting schemes,^42−45^ the DKS module of BERTHA has allowed us to benefit from the hardware developments of the last 15 years and from the new trend of prototyping and interoperability of software development. We have shown that it is possible to greatly reduce the weight of the computational burden of a DKS computation by implementing different parallelization and memory distribution schemes on different HPC architectures.^46−49^ Recently, the introduction of a Python Application Programming Interface (API) to BERTHA, which we refer as PyBERTHA,^49−52^ allowed us to easily implement the real-time TDDKS scheme to study the electrodynamics under strong fields and frozen density embedding including the environmental effects.^53^

In this work, we show that OpenACC directives (and CUDA Fortran) can be used to port the DKS module of the BERTHA code to the GPU, resulting in an efficient hybrid CPU/GPU implementation. Furthermore, we have also extended the porting to the Python API and enable the acceleration of the real-time TDDKS method on the GPU. In the latter implementation, we have used the CuPy library (instead of Numpy, which is used on the CPU) for linear algebra to evaluate the exponential form of the time evolution operator needed in the real-time propagation. Although the approach developed here is not yet optimal, it is very promising and efficient compared to the CPU-parallel shared memory version (with OpenMP and Numpy).

The implementation strategy we adopted is relatively simple and, as already mentioned, is mainly based on compiler directives. It has the advantage of maintaining a single source code without duplicates, which we expect will be easier to maintain and can be further improved in the future. The initial development of this project was carried out as part of the CINECA GPU Hackathon^54^ and all runs were performed on the Leonardo supercomputer at CINECA.

The paper is organized as follows. Section 2 gives a brief overview of the main theoretical aspects of the DKS method as implemented in BERTHA, with particular reference to the variational density fitting scheme used. We also describe the Python API and the main computational steps required to implement the real time TDDKS method (PyBERTHART). We will only focus on the most important steps that are useful for understanding offloading to the GPU. Section 3 describes our GPU-accelerated single-point SCF (Section 3.1) and real-time TDDKS (Section 3.2) programs in detail. In Section 4 we give instead all the performance in terms of speedups over the CPU versions (both serial and parallel) of the codes. Finally, we draw some conclusions in Section 5.

## 2 Theoretical Background and Implementatio

In this section, we give a brief overview of the implementation of the DKS method in the BERTHA code and its Python API extension (PyBERTHA), which is used to implement the real-time TDDKS approach (PyBERTHART). We mainly focus on the aspects that are important to fully understand our new port to the GPU. For further details, we refer the interested reader to the cited literature.^37,49^

The DKS equation is a relativistic generalization of the Kohn–Sham equation, and, considering only the longitudinal electrostatic potential, it can be written as follows
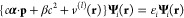
1being *c* the speed of light in vacuum, and **p** the electron momentum, while
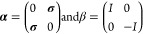
2where **σ** = (σ_*x*_, σ_*y*_, σ_*z*_), σ_*q*_ is a 2 × 2 Pauli spin matrix and *I* is a 2 × 2 identity matrix. The longitudinal interaction term (*v*^(*l*)^(**r**)) is the sum of the nuclear potential *v*_N_(**r**), the Coulomb interaction *v*_H_^(*l*)^[ρ(**r**)] and the exchange–correlation term *v*_XC_^(*l*)^[ρ(**r**)], while the Breit interaction contribution is not considered here. We express the 4c spinor solution of eq 1 as a linear combination of G-spinor basis set functions^41^ (*M*_μ_^*T*^(**r**) with *T* = *L*, *S*)
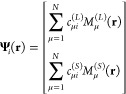
3where *L* and *S* refer to the so-called “large” and “small” components. In the G-spinor representation, we define the density matrices as follow
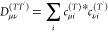
4where the sum runs over the occupied positive-energy states. The total electron density is obtained according to
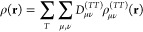
5

An important property of the G-spinor functions is that an overlap density (ρ_μν_^(*TT*)^(**r**)) can be expressed as
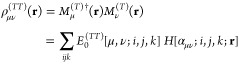
6This is a finite linear combination with the coefficients *E*_0_ of standard Hermite–Gaussian-type functions (HGTFs) *H*[α_μν_; *i*, *j*, *k*; **r**] (see e.g. ref (55)). The *E*_0_ coefficients, which contain the entire spinorial structure, are described in ref (56) and evaluated with the help of effective recurrence relations.^41^ The ranges of the summations over *i*, *j* and *k* are uniquely determined by the indices that specify the basis functions and, in particular, are related to the angular momentum quantum numbers of the two spinors involved (see ref (37) for details).

In eq 3, *c*_μ*i*_^(*T*)^ are expansion coefficients that are determined by solving the following generalized eigenvalue equation
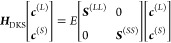
7where ***H***_DKS_ is the matrix representation of the DKS operator in the G-spinor basis, and it is defined in terms of ***v***^(*TT*)^, ***J***^(*TT*)^, ***K***^(*TT*)^, ***S***^(*TT*)^, and **Π**^(*TT*^′^)^ matrices, with *TT* = *LL*, *SS* and *TT*′ = *LS*, *SL*. These matrices are the representation of the nuclear term (the nuclear charges were modeled by a finite Gaussian distribution^57^), the Coulomb and exchange–correlation potentials, the overlap matrix and the matrix of the kinetic operator,^37,41^ respectively. It is clear that the ***H***_DKS_-matrix depends on the total electronic density ρ(**r**) due to *v*_xc_^(l)^[ρ(**r**)] and *v*_H_^(l)^[ρ(**r**)], through the canonical spinors obtained by their diagonalization. The final solutions are thus obtained in a self-consistent way. One of the most time-consuming parts of the procedure is the calculation of the Coulomb and exchange correlation contributions to the DKS matrix (i.e., the ***J***^(*TT*)^ and ***K***^(*TT*)^ matrices) together with the diagonalization of the ***H***_DKS_ itself.

However, the current version of the BERTHA code takes advantage of the density fitting techniques^42−44^ for the efficient evaluation of the ***J***^(*TT*)^ and ***K***^(*TT*)^ matrices. In particular, we use the variational density fitting scheme introduced by Köster et al.^45^ in a nonrelativistic context. Density fitting is a technique used to reduce the computational cost and scaling of DKS calculations. It involves the expansion of the relativistic density by a linear combination of atom-centered auxiliary functions, *f*_*t*_(**r**)
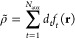
8The coefficients *d*_*t*_ are chosen to minimize the error when the fitted density is used to evaluate the Coulomb energy. This condition gives a linear system for the vector of fitting coefficients, as follows
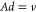
9where *A* is a real symmetric matrix representing the Coulomb interaction in the auxiliary basis (*A*_*st*_ = ⟨*f*_*s*_∥ *f*_*t*_⟩) while *v* is a vector that represents the projection of the electrostatic potential onto the fitting functions
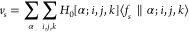
10where ⟨*f*_*s*_∥α; *i*, *j*, *k*⟩ are two-electron integrals between the auxiliary function function *f*_*s*_ and the Hermite functions of Gaussian type (HGTF) *H*[α; *i*, *j*, *k*; ]. *H*_0_[α; *i*, *j*, *k*] are coefficients resulting from the generalization of the scalar Hermite density matrix, proposed by Almlöf,^58,59^ as follows
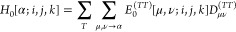
11where the second summation runs over all pairs of basis functions with the same exponent α and origin, which follows from Gaussians product theorem.^55^

A very similar strategy is adopted for the exchange–correlation **K** matrix. Where a similar linear system is solved
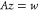
12in this case *w* is the vector resulting by the projection of the exchange–correlation potential of the fitted density  on the fitting functions
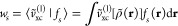
13

The vector *w*, which involve integrals of the exchange–correlation potential, is computed numerically by the integration scheme already implemented in the code.^60^ The two vectors *d* and *z* are finally used to efficiently build the Coulomb *J̃* and the exchange–correlation *K̃* matrices as a function of 3-centers two-electrons integrals *I*_*a*,μν_^(*TT*)^ = ⟨*f*_*a*_∥ρ_μν_^(*TT*)^⟩, as follows
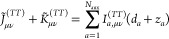
14
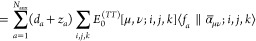
15where eq 15 is obtained by eq 14 using the definition of the G-spinor overlap density (eq 6). The efficiency of this approach depends on the choice of auxiliary basis function, and we employ the Hermite Gaussian-type functions (HGTFs) as a natural choice because they allow for the direct evaluation of two-electron integrals. The use of primitive HGTFs that are grouped together in sets sharing the same exponents further simplifies the calculations.^37,43,44^ We can schematically summarize the density fitting procedure in two steps (both scaling as O(N^3^)):

- step 1: create and solve two linear systems: *Ad* = *v* and *Az* = *w*
- step 2: evaluate  via eq 15

For the sake of completeness, we mention that in the present implementation of the DKS model in BERTHA we use exchange correlation functionals and the associated potential in the local density approximation (LDA), the generalized gradient approximation (GGA) and the meta-GGA using the so-called density only approximation (i.e., it depends on the density, its gradient or Laplacian, but not on other variables such as the spin density or the magnetization).

**Figure 1:**
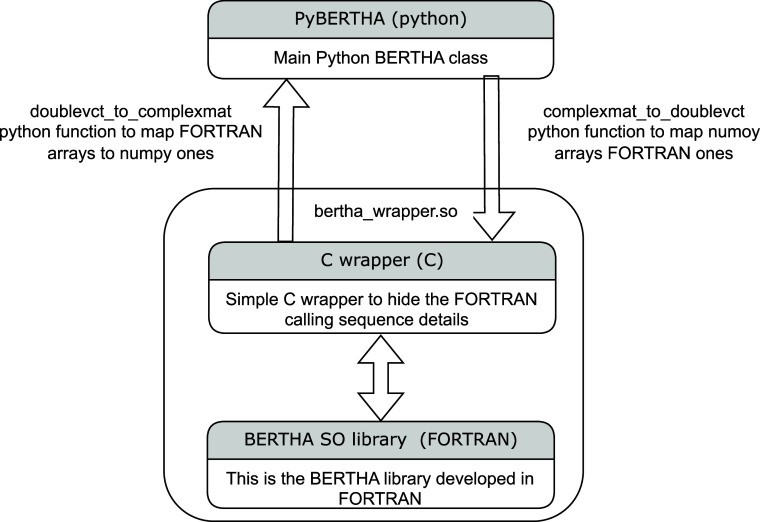
Code is involving three main software layers. At the bottom there is the FORTRAN layer, where the calculation of the H_*DKS*_ matrix is performed. On top of the FORTRAN layer there is a C wrapper that is used to simplify the interlanguage communication between the FORTRAN and Python. Two python functions have been implemented to easily map matrices from/to *numpy* arrays to/from proper FORTRAN double complex ones.

Now, we briefly present our real-time TDDFT implementation in the context of the relativistic 4c DKS theory, as developed in the BERTHA code using the recent Python API, PyBERTHA.^50,51,61^ This Python API has been described in details in ref (49) and is summarized in Figure 1.

It is made of the different layers. The basic FORTRAN kernel functions of BERTHA are collected in a single Shared Objected (SO), **libertha.so**, that can run both in serial or using OpenMP^62^ (on many cores).

On top of this we also implemented a FORTRAN module, named **bertha_wrapper**, which uses the standard intrinsic module (*iso_c_binding*) for C interoperability, and gives the possibility to access to all the basic quantities, such as energy, density, DKS and overlap matrices and other quantities. The same FORTRAN module is used also to access all BERTHA functionalities such as **bertha_init** to perform all the memory allocations, **bertha_main** to run the main SCF iterations, and **bertha_finalize** to free all the allocated memory, and more. Finally the main PyBERTHA^50,52^ module has been developed using the **ctypes** Python module. This module provides the C-compatible data types, and allows calling functions collected in shared libraries. In order to simplify the direct interlanguage communication between Python and FORTRAN, we developed a C layer called **c_wrapper**. This layer is particularly useful especially when one has to deal with different FORTRAN compilers. Indeed each FORTRAN compiler produces a different symbol name for the same subroutine. Clearly, this FORTRAN characteristic can be easily managed using the C preprocessor directives. This functionality, as it will be discussed below, has been important for the GPU porting.

In the present version of the code the input geometry, basis and fitting set need to be specified via a file and the related **set_fnameinput** and **set_fittfname** methods. Additionally the **pybertha** class is populated with all the basic functionality required to implement basic procedures as a single-point energy calculation (using the **run** and **get_etotal** methods). All desired quantities can be made available and efficiently manipulated at Python layer as Numpy arrays. More complex methods can be devised, such as, for instance, **get_realtime_fock** to obtain the DKS matrix given as an input a density matrix, which is used in the implementation of the time-dependent DKS method we will describe in the following.
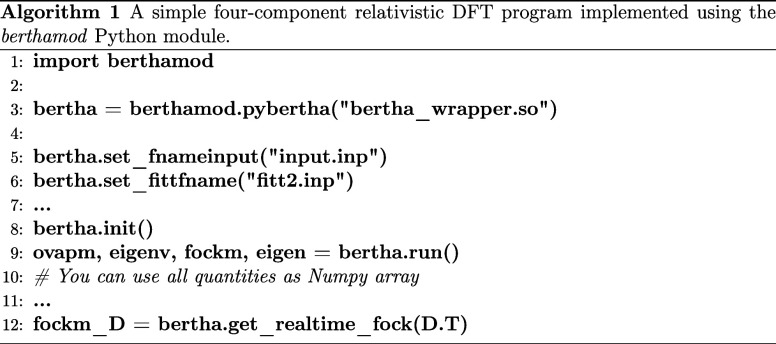


With this Python environment, the implementation of the real-time TDDKS scheme was relatively simple. In the following, we present the most important aspects of the theory, emphasizing in particular the important computational aspects. The time-dependent equation for the Hartree–Fock and density functional theory can be reformulated by the Liouville-von Neumann equation (LvN). In an orthonormal basis set, the LvN equation reads
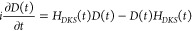
16*D*(*t*) and *H*_*DKS*_(*t*) are the one-electron density matrix and time-dependent Dirac–Kohn–Sham matrix, respectively. For the sake of simplicity, we have omitted the explicit reference to the labels (*T* = *L*, *S*) in the equations. The time dependence of *H*_DKS_(*t*) results from the explicitly time-dependent external electric field *v*_ext_(*t*) and implicitly via the time dependence of the density matrix *D*(*t*) in the Coulomb (*J*[ρ(*t*)]) and exchange correlation terms (*K*_*xc*_[ρ(*t*)]). The dynamics of the systems is described by the time evolution of the density matrix, which can be expressed as follows
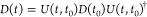
17where *U*(*t*, *t*_0_) is the matrix representation of the time-evolution operator. In the most general case, the time evolution operator is expressed a time-ordered series
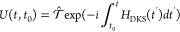
18where  is the time-ordering operator.

If the discrete time interval, Δ*t*, is chosen to be small enough the time-ordering can be ignored and the propagator may be written in the form
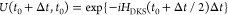
19where *U*(*t*, *t*_0_) is the matrix representation of the time-evolution operator.

The whole propagation scheme is carried out by repeatedly applying the propagator at each time step. While all the details have been reported in our previous paper,^51^ it is worth to mention here that the time propagation of the density matrix is carried out by means of a midpoint Magnus propagator, with a self-consistent predictor/corrector scheme which closely follows the strategy proposed by Repisky et al.^63,64^ In Algorithm 2 we emphasize the most important calculations required at each time step. This requires one DKS matrix evaluation, one diagonalization and eight matrix–matrix multiplications (for the transformation from the atomic to the molecular basis representation) per micro iteration of the self-consistent predictor/corrector scheme. The cycle repeats until convergence is reached. According to Repisky et al.,^63^ the convergence criterion chosen is that the Frobenius norm of the difference between density matrices obtained in the *n* and the (*n* + 1)th iterations must be lower than a given threshold (line 16) of Algorithm 2. We have implemented the entire scheme in a Python code,^51^ in which key variables can be accessed directly from the module **berthamod**, while to perform linear algebra operations (matrix–matrix multiplications and diagonalization) we can use the highly optimized methods implemented in the widely used NumPy module^65^ or, as we will emphasize below, the corresponding GPU-enabled CuPy.^22^
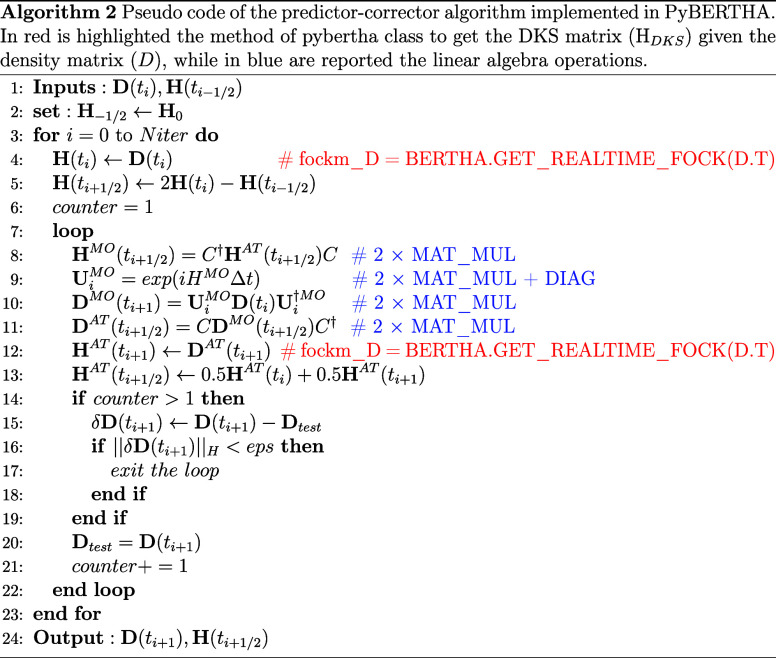


## 3 GPU Porting of the BERTHA/PyBERTHA and PyBERTHART Codes

In this section, we will report on all the details of the GPU porting of the BERTHA/PyBERTHA and PyBERTHART codes. Thus, we will describe all the details of both the single-point DKS energy calculation and the RT-TDDKS simulations.

The simple underlined structure of the FORTRAN code available in BERTHA allowed us to port the code to GPUs relatively easily, resulting in an efficient hybrid CPU/GPU (OpenACC/OpenMP) implementation, where the most computationally intensive part (three-center two-electron integrals evaluated via the McMurchie–Davidson scheme) for the DKS matrix construction is efficiently ported to the GPU via the high-level programming model OpenACC using compiler directives. The data transfer on the host device was performed using simple OpenACC directives for the compilers (e.g., !$acc& copyin(.)). This scheme in combination with the use of an optimized linear algebra library on GPUs (cuBLAS and cuSOLVER) significantly accelerates DKS calculations. Furthermore, we have used this new low-level integral kernel computation and the use of the Python module CuPy to offload our real-time propagation of time-dependent DKS (RT-TDDKS) (PyBERTHART) to the GPU.

### 3.1 BERTHA/PyBERTHA Porting: Single Point Energy DKS Calculation

To describe the GPU acceleration of the code, it is convenient to describe the main computational steps involved in a single-point DKS energy calculation in four main steps:

1. calculate all one-electron matrices that do not change from cycle to cycle (the one-electron and overlap matrices).
2. Carry out step 1 of density fitting (from now on called “density fitting”), which is the calculation and the solution of the two linear systems (eqs 9, 10, 13 and 12).
3. Carry out step 2 of density fitting and create the Coulomb and exchange correlation matrix *J* + *K* (from now on simply called “*J* + *K*”) using eq 15.
4. Assemble the *H*_DKS_ (Dirac–Kohn–Sham matrix) and solve the generalized eigenvalue problem to finally obtain a new density matrix with which you can start the next iteration. This step will be named “linear algebra” from now on, as it comprises the solution of the eigenvalue problem, the level-shift (i.e., two matrix multiplication) and the construction of the new density matrix (i.e., a matrix multiplication)

**Figure 2:**
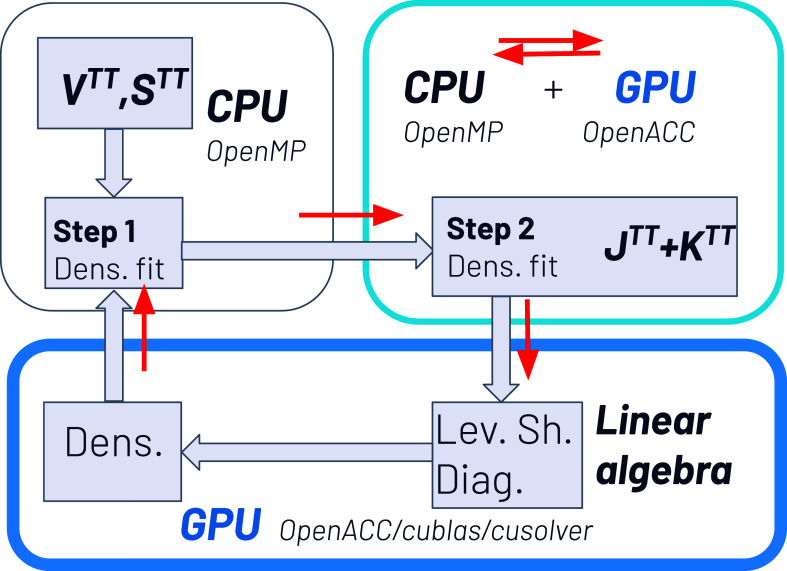
A schematic workflow of the most important steps of the calculation of the single point energy calculation. In the figure, it is underlined whether the calculation step is performed on the CPU or GPU. We also highlight when some explicit data is transferred from the CPU to the GPU or vice versa, see text for details.

Considering the above steps, we can easily see here (see Table 1 entries Serial and OpenMP) that the most demanding steps in the serial version are the steps “linear algebra” and “*J* + *K*”. Looking at the serial code, the quoted “linear algebra” step is between 67% and 79% depending on the system, and the last step, i.e. the calculation of the *J* + *K* matrix, takes 20%–24% of the total time. The relative cost of this last step tends to decrease (up to 7% for Au_16_) if we take into account the data obtained with the parallel version of the code. The basic guideline we followed was to keep the code structure as simple as possible while balancing the potential performance gain of offloading to the GPU and portability. We have taken an incremental approach to offloading and used a platform-independent high-level language for programming accelerators such as OpenACC. In a way, the OpenACC^66^ approach is significantly closer to the parallel shared memory scheme using the OpenMP approach on CPUs, in the sense that an OpenMP code is structured (or designed) to have independent loops. In this work, we focus on the GPU acceleration of the code involved in the “*J* + *K*” and “linear algebra” steps by using an incremental approach starting from the OpenMP version of the code. The approach we chose is a hybrid approach where OpenMP (on the CPU) and OpenACC (host/device data transfer and distributed computation on the GPU) were used to accelerate the code on the FORTRAN layer. Our porting strategy is summarized in Figure 2, where the gray arrows follow the SCF flow and the red arrows highlight where the data transfer between host (CPU) and device (GPU) takes place. In particular, the first two tasks of the SCF method, namely the evaluation of all one-electron matrices that do not change from cycle to cycle (the one-electron and overlap matrices) and step 1 of the density fitting are evaluated on the CPU (in serial or in parallel using OpenMP). For step 2 of the density fitting (“J + K”) we have a mixed approach in which we use the OpenACC directives to facilitate the construction of blocks of 3-center two-electron integrals of the eq 15, while OpenMP is used to distribute the evaluation of the *E*_0_^*TT*^ coefficients to the CPU cores. In practice, we need to transfer all the necessary information from the CPU to the GPU to evaluate these blocks of two-electron integrals. This includes the density fitting coefficients (vectors **d** and **z**), information about the fitting functions (exponents, origin) and information about the definition of the HGTFs resulting from the Gaussian product theorem of the G-spinor basis functions. These integrals (, see eq 15) are evaluated in parallel on the GPU and partially accumulated on the device. The work on the GPU is distributed using the OpenACC directive with a simple parallel loop collapse. As soon as this group of integrals has been evaluated, it is transferred back to the CPU. The calculation of the associated *E*_0_^*TT*^ coefficients was performed in parallel on the CPU using an OpenMP instruction parallel loop. The final Coulomb and exchange correlation matrices are thus accumulated directly on the CPU and summed to the one-electron matrix to obtain the full DKS matrix (*H*_DKS_).

**Table 1:** Time in Seconds (s) for the Calculation of the Different Steps of a Single SCF Iteration (See Text for Details) for Many Molecular Systems (Au_2_, Au_4_,Au_8_,Au_16_)^a^

	num. threads	density fitting (s)	*J* + *K* (s)	linear algebra (s)	one electron (s)	tot. time per SCF iteration (s)
Au_2_ (1632)						
serial		1.340	2.820	7.800	0.760	11.585
OpenMP	32	0.090	0.430	0.590	0.030	1.125
OpenMP/ACC	4	0.370	0.150	0.120	0.200	0.540
OpenMP/ACC	8	0.200	0.115	0.120	0.103	0.397
OpenMP/ACC	16	0.115	0.100	0.120	0.053	0.322
OpenMP/ACC	32	0.090	0.100	0.120	0.033	0.307
Au_4_ (3264)						
serial		5.475	20.395	67.425	2.890	91.790
OpenMP	32	0.280	1.970	11.260	0.130	13.540
OpenMP/ACC	4	1.485	0.600	0.515	0.760	2.235
OpenMP/ACC	8	0.775	0.460	0.500	0.393	1.592
OpenMP/ACC	16	0.430	0.400	0.495	0.207	1.283
OpenMP/ACC	32	0.280	0.390	0.495	0.130	1.165
Au_8_ (6528)						
serial		24.890	154.180	605.260	12.960	778.445
OpenMP	32	1.220	10.420	103.530	0.550	115.255
OpenMP/ACC	4	6.805	2.670	2.405	3.290	10.485
OpenMP/ACC	8	3.525	2.120	2.375	1.690	7.460
OpenMP/ACC	16	1.920	1.885	2.375	0.863	6.032
OpenMP/ACC	32	1.225	1.830	2.380	0.553	5.457
Au_16_ (13,056)						
serial		128.890	1180.220	4761.080	59.190	6045.770
OpenMP	32	6.350	62.100	824.790	2.740	893.680
OpenMP/ACC	4	35.740	13.220	14.595	16.207	57.933
OpenMP/ACC	8	18.650	11.100	14.580	8.320	42.105
OpenMP/ACC	16	10.050	10.215	14.625	4.307	34.368
OpenMP/ACC	32	6.410	10.015	14.735	2.737	31.398

a In parentheses is reported the dimension of the associated DKS matrix. Data was obtained with the CPU version in a single core (Serial), with multiple cores using OpenMP (OpenMP) and with the new hybrid CPU/GPU implementation (OpenMP/ACC). The data was obtained on an Intel Xeon Platinum 8358 CPU with 2.6 GHz (32 cores) using the NVIDIA HPC compiler (nvFortran) with the same compilation flags for CPU and CPU/GPU (-Mextend -O3) and the multithreaded openblas library. The results were obtained on the new Tier-0 EuroHPC supercomputer (LEONARDO) at CINECA. For GPU-accelerated data (OpenMP/ACC) we use a single NVIDIA A100 GPU. See text for details.

The GPU acceleration of the code then focused on the “linear algebra” operations (field Figure 2). Porting this part was relatively easy thanks to calling CUDA library functions from NVIDIA Fortran with OpenACC and its compatibility with CUDA Fortran. In practice, we performed the following operations:

- transfer the density matrix, *H*_DKS_ and the overlap matrices from the CPU to the GPU using OpenACC directives (e.g., !$acc data!$acc data···)
- perform the level shift (i.e., two zhemm calls executed by the OpenACC host code using the use cublas module).
- Diagonalise the H_*DKS*_ matrix (i.e., using the cusolverDnZhegvd with CUDA FORTRAN interface).
- Calculate the new density matrix (i.e., using the function zgemm from the OpenACC host code with the use cublas module).
- Transfer the eigenvectors, eigenvalues and density matrices back to the CPU. This happens automatically when the OpenACC region is closed (!$acc end data).

A look at the data shown schematically in Figure 4, it becomes clear that the bandwidth from the CPU to/from the GPU is a potential bottleneck in the GPU acceleration of the code, albeit mainly in the form of communication latency. To minimize the communication between CPU and GPU, we first combined the level shift (two matrix multiplications), the diagonalization and the construction of the new density matrix (one matrix multiplication) into a single function that is executed on the GPU. Of course, we could avoid transferring the overlap matrix at each iteration and also transfer some data from the GPU back to the CPU to further optimize the code. However, in the current implementation we have preferred to keep the code as simple as possible, also considering that the mentioned data transfer operations (i.e., mainly the transfer of the overlap matrix from the CPU to the GPU at each iteration) have a negligible impact on the overall time, as we will clearly see looking at the final results in terms of speedup in the next section.

**Figure 3:**
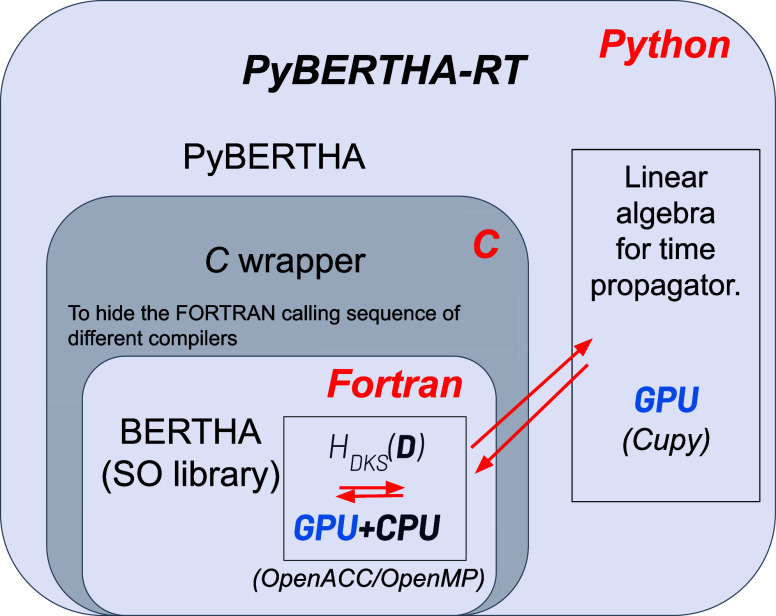
We are summarizing here the various software layers of our RT-TDDKS code, underlying also where the GPU acceleration is taking place.

**Figure 4:**
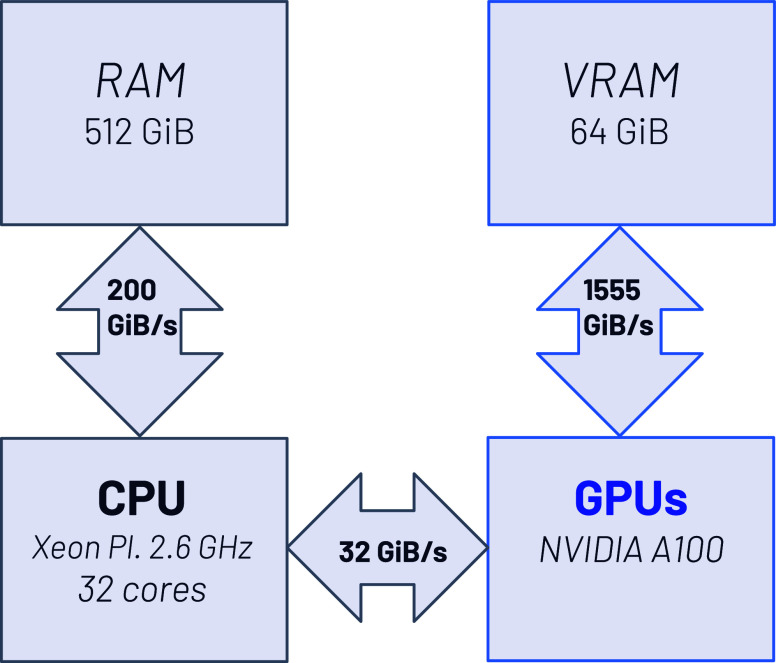
A schematic representation of the essential elements of the individual node architectures of the pre-exascale Tier-0 EuroHPC supercomputer LEONARDO at CINECA, focusing on the bandwidth between the components.

We conclude the present subsection by citing a fairly straightforward step, considering our open-ended parallel MPI implementation of the BERTHA code,^46−48,50^ it is a multi-GPU extension of the current version. Concretely, in the MPI version of our code we found that a simple and efficient parallel construction of the *J* and *K* matrices can be achieved by cyclically assigning to each process the allocation and computation of blocks whose offsets and dimensions depend on the specific structure of the G-spinor matrices. Quite easily each process could take advantage of a GPU to further speedup each *J* + *K* block calculation. Concerning instead the “linear algebra” step we could take advantage of some of the already available Multiprocess and multi-GPU ScaLAPACK-like libraries such as ELPA^67,68^ or the cuSOLVEMp^69^ one.

### 3.2 PyBERTHART Porting: Real-Time TDDKS

We have already summarized the main computational steps required to implement the real-time TDDKS scheme in PyBERTHART (see Algorithm 2). Here we highlight the aspects that are useful to understand the key points of our porting on devise. First, it is important to remember that the code consists of three main software layers (see Figure 3). At the bottom is the FORTRAN layer, where the calculation of the *H*_DKS_ matrix is performed. Above the FORTRAN layer is a C wrapper, which is used to simplify the FORTRAN-Python interlanguage communication. The C wrapper has proven to be particularly useful here, as the NVIDIA FORTRAN compiler, which is based on the Portland compiler,^70^ has a completely different naming convention both for data and subroutines/functions than the previously used compilers, namely Intel and GNU. Without the cited wrapper, the Python layer code would have been severely affected by this port. Finally, there is the Python layer, on which the most important calculation steps for real-time propagation are executed.

As already mentioned, real-time propagation (see also Algorithm 2) involves the calculation of the H_*DKS*_ using the updated density matrix at each time step and a series of linear algebra operations. In Figure 3 we schematically illustrate where (on the host or on the device) different steps are carried out and the key points at which the computational flow requires data between the host and the device and vice versa. This should help the reader to fully understand the computational steps described and the results we obtained.

In the current version, the *H*_DKS_ matrix is evaluated at the FORTRAN layer using the hybrid CPU/GPU approach developed here (OpenMP/OpenACC), as explained in the previous section, while the density matrix is propagated in time through a series of linear algebra operations (originally performed with Numpy) and is now offloaded to the GPU via Cupy.

Our porting can be briefly summarized by the following main steps:

- the transfer of the data (i.e., the initial density matrix and the *H*_DKS_ matrices as well as the overlap and eigenvector matrices) from the CPU to the GPU memory is required to start the time propagation (see line 1 of the algorithm 2). These matrices are in fact generated in the FORTRAN layer using the hybrid CPU/GPU (OpenMP/ACC) implementation.
- The time propagation of the density matrix is carried out at Python level (line 3 of the Algorithm 2) with two nested loops. During each time step, there is a loop over microiterations (line 7) associated with the predictor/corrector algorithm. This involves: (i) many linear algebra operations, including matrix multiplications and a matrix exponential (line 7) of the *H*_DKS_ matrix (see eq 19). All these linear algebra operations (originally performed with Numpy) are now offloaded to the GPU via Cupy. and (ii) the calculation of the new *H*_DKS_ matrix (line 12).

In terms of data transfer, each microiteration requires data exchange between CPU and GPU. In fact, the density matrix must be transferred from the GPU to the CPU in order to calculate the corresponding *H*_DKS_ matrix, associated *H*_DKS_ matrix (i.e., to be calculated within the FORTRAN layer), which is then transferred back to the GPU. It is clear from Section 3.1 that the actual computation of *H*_DKS_ occurring at the FORTRAN layer takes place both on the CPU and on the GPU with the associated data communication. Nevertheless, we will show, in the next section, that the impact of CPU to/from GPU communication it is almost negligible.

## 4 Results and Discussions

In this section, we will report and discuss all the results in terms of speedup for both the single-point DKS energy calculation and the RT-TDDKS simulations of the GPU-accelerated version compared to the CPU-only run. All tests were performed with the Tier-0 EuroHPC supercomputer LEONARDO at CINECA^71^ running on Intel Xeon Platinum (PI) 8358 CPU with 2.6 GHz (total 32 cores). In all cases, we use the NVIDIA HPC compiler (nvFortran) with the same compilation flags for CPU and CPU/GPU (-r8 -Mextend -O3) and the same multithreaded openblas library with many cores (openblas/0.3.9-gnu-8.4.0). In the case of the accelerated CPU/GPU version we use a single NVIDIA A100 GPU and the cuBLAS and cuSOLVER libraries for GPU-accelerated linear algebra operations (-acc = gpu -gpu = cc80,cuda11.8 -Minfo = accel -cuda -cudalib = cublas,cusolver). In Figure 4 we summarize the essential elements of the individual node architectures, focusing mainly on the bandwidth of the various system buses. The figure makes it clear that an important point when porting codes to GPUs is to minimize the data transfer between host (CPU) and device (GPU).

To prove the effectiveness of our approach, we consider a series of gold clusters with increasing size from Au_2_ to Au_16_. For the Au atom, the basis set for the large component was generated by uncontracting double-ζ quality from the Dyall basis set (dyall.v2z),^72^ while the corresponding basis for the small component was generated using the restricted kinetic balance relation.^39^ For gold, the optimized B16 auxiliary basis sets were used.^43^ The geometries were given in Supporting Information for all systems.

### 4.1 Single Point Energy DKS Calculation

In Table 1 we give the timing for the different steps of the SCF cycle using the new CPU/GPU-accelerated (OpenACC/OpenMP) version of the DKS module of the BERTHA code. For reference, the same table also shows the results obtained with the serial and parallel OpenMP multithreaded versions using 32 cores running exclusively on the CPU.

**Figure 5:**
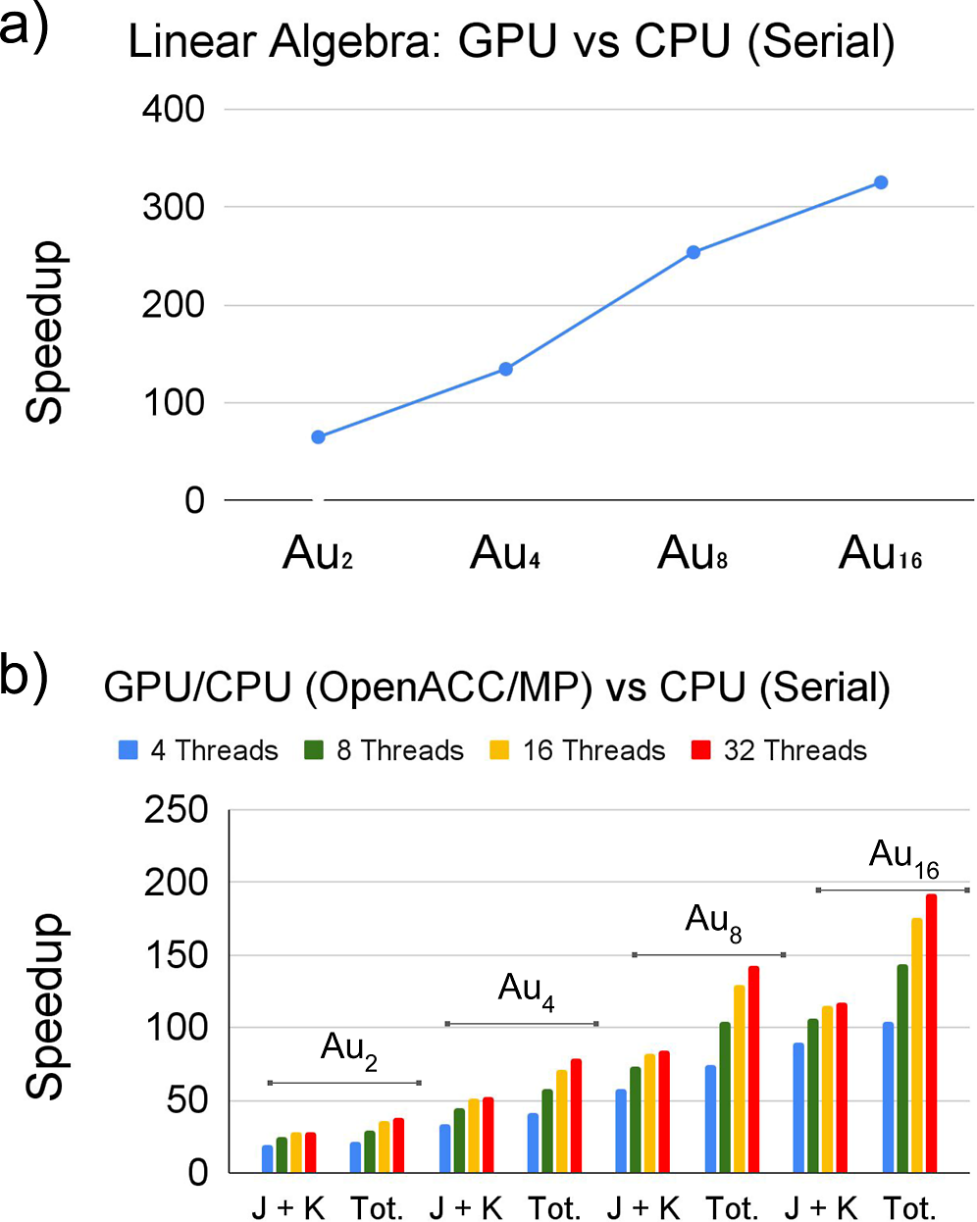
(a) We give the total time for the “linear algebra” step as a function of the system size. (b) We report the acceleration of the “*J* + *K*” and the SCF “iteration” compared to the serial version of the code, taking into account different number of threads.

The results in terms of acceleration compared to the serial CPU run are shown in Figure 5. The speedup of the “Linear Algebra” step is enormous when compared to the serial version (i.e., it ranges from 65 to more than 300 times faster for the Au_2_ and Au_16_, respectively). Interestingly, while this linear algebra step scales as expected with O(N^3^) for the serial and parallel multithreaded computation on the CPU (see Table 1) with the system size, the scaling is significantly smaller for the computation on the GPU O(N^2.5^). This indicates that GPU architectures are utilized more efficiently in larger systems.

The speedup of the “*J* + *K*” step of the hybrid CPU/GPU (OpenACC/OpenMP) code using a different number of threads, which can be seen in Figure 5 (panel b). The GPU acceleration here is also very satisfactory. And again it increases with the system size. We gain a speedup ranges from 19 for Au_2_ to 91 for Au_16_ when using 4 threads in the hybrid OpenACC/OpenMP code. Interestingly, the speedup increases only slightly with the number of cores used (the speedup when using 32 threads is 28 and 118, respectively), this is because the speedup is mainly driven by the GPU.

**Figure 6:**
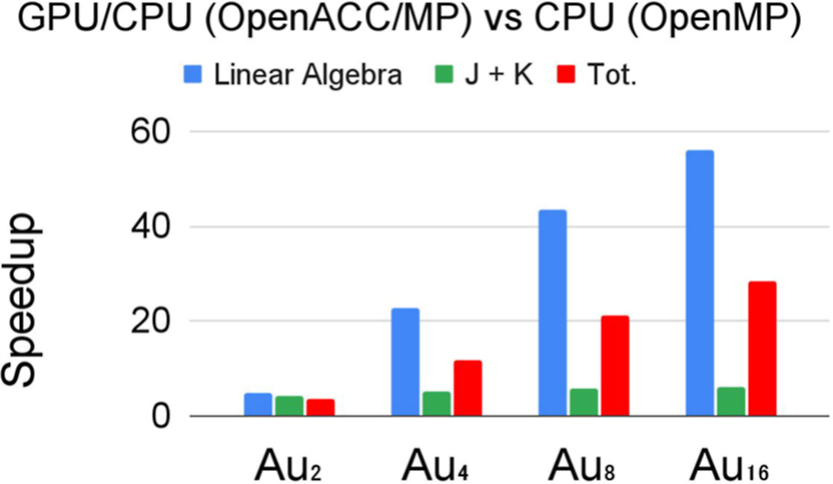
Speedup of the wall time for the “linear algebra” step, *J* + *K* and for the total time using 32 threads.

In Figure 6 we show the speedup of the GPU-accelerated version compared to the multithreaded OpenMP (32 threads) running exclusively on the CPU (i.e., we use the same number of threads in both versions of the code). The overall performance is very satisfactory. The speedup at the “linear algebra” step is between 5 and 56 times, while at the “*J* + *K*” step we can observe a speedup of 4.3 to 6.2 times for Au_2_ and Au_16_ respectively. The total acceleration of the complete SCF iteration also increases with the system size and ranges from 4 to 29.

### 4.2 Real-Time TDDKS: PyBERTHART on GPU

In Table 2 we give the average time of the main phases of the calculation for a series of molecules with an increasing size (namely gold clusters from Au_2_ to Au_8_ and a water molecule) using the hybrid CPU/GPU version with CuPy (Cupy,OpenMP/ACC) compared to the CPU version and Numpy with 32 threads (Numpy,OpenMP). Data are given for the calculation of the *H*_DKS_ matrix, the matrix exponential and for the full RT-TDDKS time step propagation as well as for the total CPU-to/from CPU communication time. The evaluation of the “matrix exponential” and the *H*_DKS_ matrix are the two most demanding operations. Indeed, they account for almost 80% of the total time, as they are performed several times during the time propagation. In general, the number of micro iterations in the predictor-corrector algorithm depends on a certain convergence threshold for the density matrix (usually between two and eight). For simplicity, we have set this number to six for all test calculations reported in Table 2.

**Table 2:** Time in Seconds (s) for the Calculation of the Different Steps of a Real-Time Micro-Iteration (See Text for Details) for Many Molecular Systems (Water, Au_2_, Au_4_ and Au_8_)^a^

	num. of threads	compute *H*_DKS_ avg. time (s)	matrix exp. avg. time (s)	CPU to/from GPU comm. (s)	total time (s)
H_2_O					
serial		0.382	0.015		2.831
NumPy, OpenMP	32	0.101	0.017		0.862
CuPy, OpenMP/ACC	4	0.101	0.002	0.019	0.788
CuPy, OpenMP/ACC	8	0.062	0.001	0.019	0.494
CuPy, OpenMP/ACC	16	0.043	0.002	0.018	0.363
CuPy, OpenMP/ACC	32	0.038	0.002	0.018	0.316
Au_2_					
serial		4.601	6.503		88.981
NumPy, OpenMP	32	0.680	0.663		11.686
CuPy, OpenMP/ACC	4	0.726	0.048	0.232	5.653
CuPy, OpenMP/ACC	8	0.487	0.048	0.231	3.979
CuPy, OpenMP/ACC	16	0.370	0.048	0.230	3.163
CuPy, OpenMP/ACC	32	0.341	0.048	0.232	2.951
Au_4_					
NumPy, OpenMP	32	3.043	11.724		105.600
CuPy, OpenMP/ACC	4	4.694	0.286	1.310	35.844
CuPy, OpenMP/ACC	8	3.090	0.276	1.317	24.535
CuPy, OpenMP/ACC	16	2.326	0.276	1.301	19.075
CuPy, OpenMP/ACC	32	2.068	0.276	1.299	17.272
Au_8_					
NumPy, OpenMP	32	15.444	107.718		834.398
CuPy, OpenMP/ACC	4	22.623	1.545	7.358	175.300
CuPy, OpenMP/ACC	8	14.693	1.549	7.350	119.638
CuPy, OpenMP/ACC	16	10.770	1.544	7.339	91.976
CuPy, OpenMP/ACC	32	9.221	1.540	7.336	80.734

a Data were obtained with the CPU version in a single core (Serial), with multiple cores using OpenMP in combination with Numpy and with the new hybrid CPU/GPU implementation in combination with Cupy (Cupy,OpenMP/ACC). The total time for a single time step of the time evolution scheme is also given (the data was obtained by setting the number of micro iterations of the predictor/corrector algorithm to six, see text for details).

The results in terms of speedup are summarized in Figure 8, where we show the speedup for the hybrid CPU/GPU version of the code compared to the CPU version using CuPy (Cupy,OpenMP/ACC) and Numpy with 32 threads (Numpy,OpenMP). The GPU acceleration of the “matrix exponential” ranges from 10, for the water molecule, to 70, when the largest gold cluster (Au_8_) is considered. If we take into account the full time step propagation, we find that the GPU-accelerated version works between 3 and 10 times faster for the water molecule and Au_8_, respectively. In the latter case, for example, the computational cost for a single time step propagation of the RT-TDDKS method decreases from 834 to 81 s. Considering that one typically needs more than 15,000 time steps for a real-time simulation, this means that your new GPU-accelerated implementation makes it possible to compute systems like Au_8_ in 2 weeks instead of 5 months. The communication time between CPU and GPU (see Figure 9) is always less than 9% of the total time (i.e., it is between 5.8% and 9.0% of the total time). Looking at the series Au_2_, Au_4_, Au_8_, we found that the communication time increases with O(N^2.5^), which is slightly higher than the expected O(N^2^).

**Figure 7:**
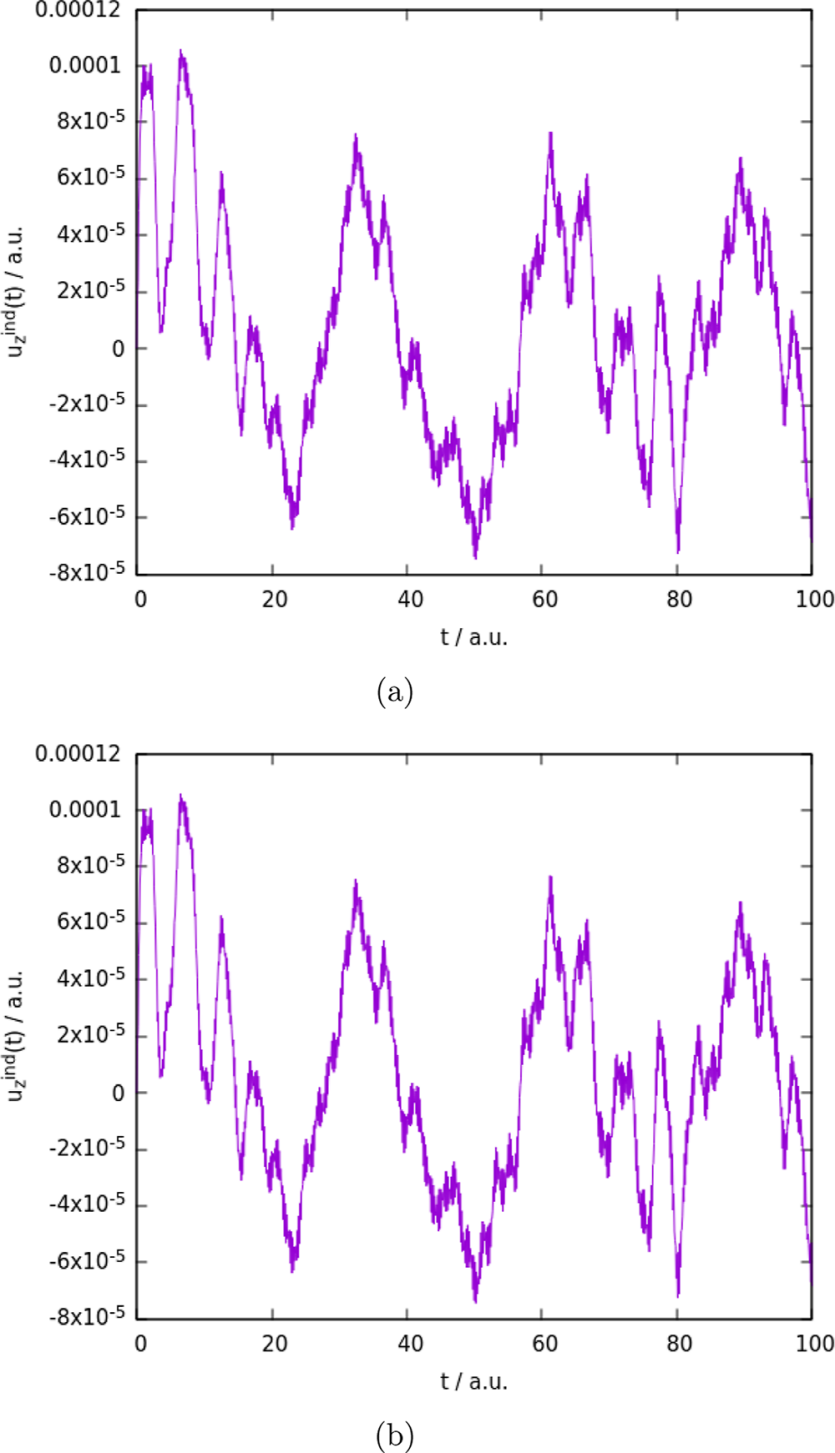
Induced dipole moment for Au_2_ obtained using the RT-TDDKS approach based on DKS method using PyBERTHA, (a) using the Cupy,OpenaMP/ACC code run on CPU/GPU (b) using the OpenMP run only on CPU (in both cases 32 CPU threads have been used.).

**Figure 8:**
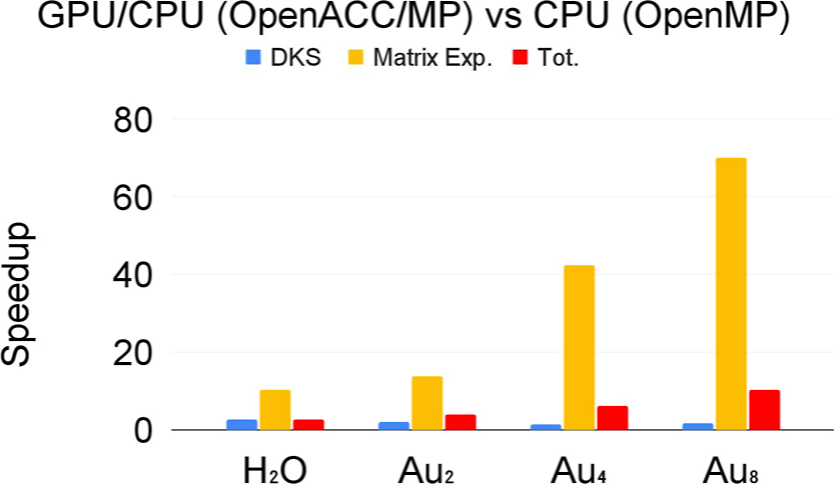
Speedup of the OpenaACC/MP code with respect to the OpenMP one using 32 threads.

**Figure 9:**
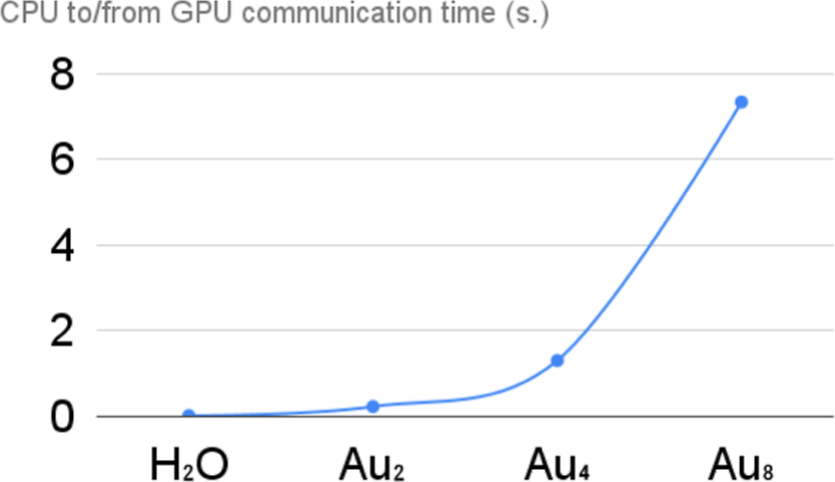
Communication time from/to CPU and GPU in the new Cupy,OpenaMP/ACC PyBERTHART code.

We also evaluated the numerical precision of the code by comparing the results obtained with the serial code with those of the GPU/CPU version. Specifically, we have carried out a real-time TDDKS simulation for Au_2_ molecule for 1000 time steps (i.e., with a time step of 0.1 au and a total time of 100 au). The results confirm the absolute numerical stability of our CPU/GPU code; indeed, the maximum difference in the induced dipole moment between the two versions is absolutely negligible (i.e., of the order of 10^–7^%) and of the same order of magnitude as the numerical precision we observe with our OpenMP code implementation (see Figure 7).

**Figure 10:**
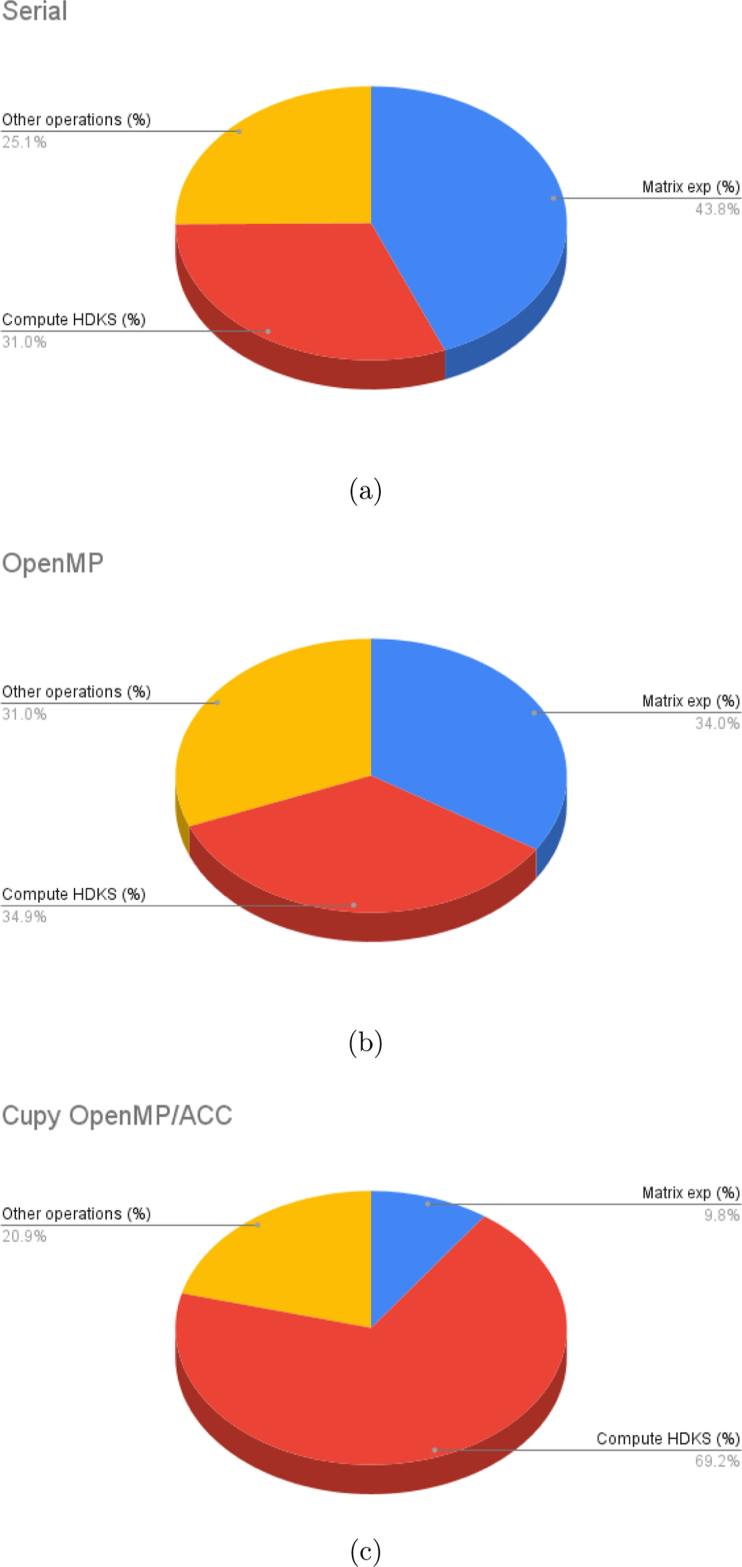
Wall time percentage of the various phases involved in the RT-TDDKS calculation for the Au_2_ molecular system: (a) Serial code (b) OpenMP code (c) Cupy OpenMP/ACC code.

Before concluding, it is also interesting to evaluate the overall impact of the GPU porting of the code on the computational burden of the various phases of the RT-TDDKS calculation. In Figure 10 we indicate the percentage of wall time for the three main phases: the “matrix exponential calculation”, the “calculation of the *H*_DKS_ matrix” and all other operations of linear algebra (i.e., indicated as “other operations”). We can notice that the “matrix exponential” and the “calculation of the *H*_DKS_ matrix” are the two most demanding phases, accounting for about 75% of the total time in both the serial and OpenMP versions of the code, with the “matrix exponential” being the most demanding one. On the other hand, looking at the Cupy, OpenMP/ACC version of the code, the “matrix exponential” phase is the less demanding phase, accounting for less than 10% of the total time, which is due to the GPU acceleration mainly related to the linear algebra operations, while the calculation of the *H*_DKS_ matrix accounts for 70% of the total time, the latter being indeed not fully ported to the GPU. This shows that there is further room for improvement if we can achieve the full porting of this step to the GPU^1^. However, this would require significant restructuring of the code and the use of lower level programming languages such as CUDA Fortran, leading to some replication of the code and making the resulting code less portable and more difficult to manage. We mention that the intermediate software layer has a low overhead due to interoperability (less than 0.05% for the serial version, less than 2 s for Au_8_^50,51^), which starts to become significant in the current Cupy,OpenMP/ACC version of PyBERTHART. Other strategies to further improve efficiency could go in the direction of using reduced Hamiltonians as exact two-component schemes or, for small/medium sized systems, using an approach where the three-center two-electron integrals are evaluated and stored in a distributed memory.

## 5 Conclusions

In this article, we have presented the latest progress in GPU acceleration of the DKS module of the BERTHA code. The DKS module of BERTHA has allowed us to benefit from the hardware developments of the last 15 years and from the new trend of prototyping and interoperability of software development. These include, for example, the open-ended memory distribution schemes of parallelization,^46,48,50^ and the Python module (PyBERTHA),^50−52,61^ which represented a significant leap forward in terms of the applicability and usability of the DKS method.

We have shown that thanks to the simple underlined structure of the FORTRAN code available in BERTHA, we were able to port the code to GPUs relatively easily, resulting in an efficient hybrid CPU/GPU (OpenACC/OpenMP) implementation, where the most computationally intensive part (three-center two-electron integrals evaluated via the McMurchie–Davidson scheme, which can handle arbitrary-order momentum basis functions) for DKS matrix construction is efficiently ported to the GPU by using compiler directives with the OpenACC high-level programming model. This scheme in combination with the use of an optimized linear algebra library on GPUs (cuBLAS and cuSOLVER) significantly speeds up DKS computations. We show that the computation of the total energy for the large Au_16_ cluster was accelerated by a factor of 30 compared to the parallel CPU implementation with 32 OpenMP threads. Furthermore, we used this new low-level integral kernel running on CPU/GPU in combination with the Python API PyBERTHA to enable a full port of the RT-TDDKS method with CuPY module for linear algebra. The results show GPU acceleration is between 3× and 10× compared to the multithreaded CPU code.

To our knowledge, this is the first report of GPU acceleration of a full 4-component relativistic code and clearly demonstrates the potential of this strategy to increase the efficiency of software for relativistic quantum chemistry. The port of the Python API (PyBERTHART) presented here is also very original and, to our knowledge, also represents the first port of a Python API to GPUs based on a FORTRAN kernel for the evaluation of two-electron integrals. Here, the main goal was to increase the speed on small to medium molecular systems for a real-time evolution scheme, but due to the simplicity of the code and the use of an open-ended parallel MPI implementation, one can easily imagine extending our code to multiple nodes and multiple GPUs.

## Supporting Information Available

This will usually read something like: “Experimental procedures and characterization data for all new compounds. The class will automatically add a sentence pointing to the information on-line (PDF)

## Footnotes

1 We can avoid transferring some data at each iteration, but we have found that if the code is compiled as a shared object (i.e., as a dynamic library) to be used by the Python code as an external library (i.e., via ctypes), the use of the device_update directive does not work properly, although this issue may have been resolved recently.

## References

[ref1] Top500 list June 2024. https://www.top500.org/lists/top500/2024/06/(accessed June, 2024).

[ref2] Yasuda K. Two-electron integral evaluation on the graphics processor unit. J. Comput. Chem. 2008, 29, 334–342. 10.1002/jcc.20779. 17614340

[ref3] Ufimtsev I. S.; Martínez T. J. Quantum Chemistry on Graphical Processing Units. 1. Strategies for Two-Electron Integral Evaluation. J. Chem. Theory Comput. 2008, 4, 222–231. 10.1021/ct700268q. 26620654

[ref4] Needham P.; Götz A. W.; Walker R. C. Electronic Structure Calculations on Graphics Processing Units; John Wiley & Sons, Ltd, 2016; Vol. Chapter 1, pp 1–22.

[ref5] Kussmann J.; Ochsenfeld C. Hybrid CPU/GPU Integral Engine for Strong-Scaling Ab Initio Methods. J. Chem. Theory Comput. 2017, 13, 3153–3159. 10.1021/acs.jctc.6b01166. 28636392

[ref6] Seritan S.; Bannwarth C.; Fales B. S.; Hohenstein E. G.; Isborn C. M.; Kokkila-Schumacher S. I. L.; Li X.; Liu F.; Luehr N.; Snyder J. W. Jr.; Song C.; Titov A. V.; Ufimtsev I. S.; Wang L.-P.; Martínez T. J. TeraChem: A graphical processing unit-accelerated electronic structure package for large-scale ab initio molecular dynamics. Wiley Interdiscip. Rev.:Comput. Mol. Sci. 2021, 11, e1494 10.1002/wcms.1494.

[ref7] Franzke Y. J.; Holzer C.; Andersen J. H.; Begušić T.; Bruder F.; Coriani S.; Della Sala F.; Fabiano E.; Fedotov D. A.; Fürst S.; Gillhuber S.; Grotjahn R.; Kaupp M.; Kehry M.; Krstić M.; Mack F.; Majumdar S.; Nguyen B. D.; Parker S. M.; Pauly F.; Pausch A.; Perlt E.; Phun G. S.; Rajabi A.; Rappoport D.; Samal B.; Schrader T.; Sharma M.; Tapavicza E.; Treß R. S.; Voora V.; Wodyński A.; Yu J. M.; Zerulla B.; Furche F.; Hättig C.; Sierka M.; Tew D. P.; Weigend F. TURBOMOLE: Today and Tomorrow. J. Chem. Theory Comput. 2023, 19, 6859–6890. 10.1021/acs.jctc.3c00347. 37382508 PMC10601488

[ref8] Barca G. M. J.; Bertoni C.; Carrington L.; Datta D.; De Silva N.; Deustua J. E.; Fedorov D. G.; Gour J. R.; Gunina A. O.; Guidez E.; Harville T.; Irle S.; Ivanic J.; Kowalski K.; Leang S. S.; Li H.; Li W.; Lutz J. J.; Magoulas I.; Mato J.; Mironov V.; Nakata H.; Pham B. Q.; Piecuch P.; Poole D.; Pruitt S. R.; Rendell A. P.; Roskop L. B.; Ruedenberg K.; Sattasathuchana T.; Schmidt M. W.; Shen J.; Slipchenko L.; Sosonkina M.; Sundriyal V.; Tiwari A.; Galvez Vallejo J. L.; Westheimer B.; Włoch M.; Xu P.; Zahariev F.; Gordon M. S. Recent developments in the general atomic and molecular electronic structure system. J. Chem. Phys. 2020, 152, 154102 10.1063/5.0005188. 32321259

[ref9] Zahariev F.; Xu P.; Westheimer B. M.; Webb S.; Galvez Vallejo J.; Tiwari A.; Sundriyal V.; Sosonkina M.; Shen J.; Schoendorff G.; Schlinsog M.; Sattasathuchana T.; Ruedenberg K.; Roskop L. B.; Rendell A. P.; Poole D.; Piecuch P.; Pham B. Q.; Mironov V.; Mato J.; Leonard S.; Leang S. S.; Ivanic J.; Hayes J.; Harville T.; Gururangan K.; Guidez E.; Gerasimov I. S.; Friedl C.; Ferreras K. N.; Elliott G.; Datta D.; Cruz D. D. A.; Carrington L.; Bertoni C.; Barca G. M. J.; Alkan M.; Gordon M. S. The General Atomic and Molecular Electronic Structure System (GAMESS): Novel Methods on Novel Architectures. J. Chem. Theory Comput. 2023, 19, 7031–7055. 10.1021/acs.jctc.3c00379. 37793073

[ref10] Manathunga M.; Miao Y.; Mu D.; Götz A. W.; Merz K. M. J. Parallel Implementation of Density Functional Theory Methods in the Quantum Interaction Computational Kernel Program. J. Chem. Theory Comput. 2020, 16, 4315–4326. 10.1021/acs.jctc.0c00290. 32511916

[ref11] Manathunga M.; Aktulga H. M.; Götz A. W.; Merz K. M. J. Quantum Mechanics/Molecular Mechanics Simulations on NVIDIA and AMD Graphics Processing Units. J. Chem. Inf. Model. 2023, 63, 711–717. 10.1021/acs.jcim.2c01505. 36720086

[ref12] Carnimeo I.; Affinito F.; Baroni S.; Baseggio O.; Bellentani L.; Bertossa R.; Delugas P. D.; Ruffino F. F.; Orlandini S.; Spiga F.; Giannozzi P. Quantum ESPRESSO: One Further Step toward the Exascale. J. Chem. Theory Comput. 2023, 19, 6992–7006. 10.1021/acs.jctc.3c00249. 37523670 PMC10601483

[ref13] Tancogne-Dejean N.; Oliveira M. J. T.; Andrade X.; Appel H.; Borca C. H.; Le Breton G.; Buchholz F.; Castro A.; Corni S.; Correa A. A.; De Giovannini U.; Delgado A.; Eich F. G.; Flick J.; Gil G.; Gomez A.; Helbig N.; Hübener H.; Jestädt R.; Jornet-Somoza J.; Larsen A. H.; Lebedeva I. V.; Lüders M.; Marques M. A. L.; Ohlmann S. T.; Pipolo S.; Rampp M.; Rozzi C. A.; Strubbe D. A.; Sato S. A.; Schäfer C.; Theophilou I.; Welden A.; Rubio A. Octopus a computational framework for exploring light-driven phenomena and quantum dynamics in extended and finite systems. J. Chem. Phys. 2020, 152, 124119 10.1063/1.5142502. 32241132

[ref14] Sharma A.; Metere A.; Suryanarayana P.; Erlandson L.; Chow E.; Pask J. E. GPU acceleration of local and semilocal density functional calculations in the SPARC electronic structure code. J. Chem. Phys. 2023, 158, 204117 10.1063/5.0147249. 37249229

[ref15] Kim I.; Jeong D.; Weisburn L. P.; Alexiu A.; Van Voorhis T.; Rhee Y. M.; Son W.-J.; Kim H.-J.; Yim J.; Kim S.; Cho Y.; Jang I.; Lee S.; Kim D. S. Very-Large-Scale GPU-Accelerated Nuclear Gradient of Time-Dependent Density Functional Theory with Tamm–Dancoff Approximation and Range-Separated Hybrid Functionals. J. Chem. Theory Comput. 2024, 20, 9018–9031. 10.1021/acs.jctc.4c01003. 39373529

[ref16] Asadchev A.; Valeev E. F. High-Performance Evaluation of High Angular Momentum 4-Center Gaussian Integrals on Modern Accelerated Processors. J. Phys. Chem. A 2023, 127, 10889–10895. 10.1021/acs.jpca.3c04574. 38090753

[ref17] Asadchev A.; Valeev E. F. Memory-Efficient Recursive Evaluation of 3-Center Gaussian Integrals. J. Chem. Theory Comput. 2023, 19, 1698–1710. 10.1021/acs.jctc.2c00995. 36917186

[ref18] Alkan M.; Pham B. Q.; Hammond J. R.; Gordon M. S. Enabling Fortran Standard Parallelism in GAMESS for Accelerated Quantum Chemistry Calculations. J. Chem. Theory Comput. 2023, 19, 3798–3805. 10.1021/acs.jctc.3c00380. 37343236

[ref19] Hammond J. R.; Deakin T.; Cownie J.; McIntosh-Smith S. Benchmarking Fortran DO CONCURRENT on CPUs and GPUs Using BabelStream. In 2022 IEEE/ACM International Workshop on Performance Modeling, Benchmarking and Simulation of High Performance Computer Systems (PMBS), 2022, pp 82–99.

[ref20] Caplan R. M.; Stulajter M. M.; Linker J. A.; Larkin J.; Gabb H. A.; Su S.; Rodriguez I.; Tschirhart Z.; Malaya N. Portability of Fortran’s ‘do concurrent’ on GPUs. arXiv 2024, 2408.07843 10.48550/arXiv.2408.07843.

[ref21] Alkan M.; Pham B. Q.; Del Angel Cruz D.; Hammond J. R.; Barnes T. A.; Gordon M. S. LibERI—A portable and performant multi-GPU accelerated library for electron repulsion integrals via OpenMP offloading and standard language parallelism. J. Chem. Phys. 2024, 161, 082501 10.1063/5.0215352. 39171700

[ref22] Okuta R.; Unno Y.; Nishino D.; Hido S.; Loomis C. CuPy: A NumPy-Compatible Library for NVIDIA GPU Calculations. In Proceedings of Workshop on Machine Learning Systems (LearningSys) in The Thirty-first Annual Conference on Neural Information Processing Systems; NIPS, 2017.

[ref23] Kriebel M. H.; Tecmer P.; Gałyńska M.; Leszczyk A.; Boguslawski K. Accelerating Pythonic Coupled-Cluster Implementations: A Comparison Between CPUs and GPUs. J. Chem. Theory Comput. 2024, 20, 1130–1142. 10.1021/acs.jctc.3c01110. 38306601 PMC10867805

[ref24] Boguslawski K.; Brzęk F.; Chakraborty R.; Cieślak K.; Jahani S.; Leszczyk A.; Nowak A.; Sujkowski E.; Świerczyński J.; Ahmadkhani S.; Kędziera D.; Kriebel M. H.; Żuchowski P. S.; Tecmer P. PyBEST: Improved functionality and enhanced performance. Comput. Phys. Commun. 2024, 297, 109049 10.1016/j.cpc.2023.109049.

[ref25] Sun Q.; Berkelbach T. C.; Blunt N. S.; Booth G. H.; Guo S.; Li Z.; Liu J.; McClain J. D.; Sayfutyarova E. R.; Sharma S.; Wouters S.; Chan G. K.-L. PySCF: the Python-based simulations of chemistry framework. Wiley Interdiscip. Rev.:Comput. Mol. Sci. 2018, 8, e1340 10.1002/wcms.1340.

[ref26] Smith D. G. A.; Burns L. A.; Sirianni D. A.; Nascimento D. R.; Kumar A.; James A. M.; Schriber J. B.; Zhang T.; Zhang B.; Abbott A. S.; Berquist E. J.; Lechner M. H.; Cunha L. A.; Heide A. G.; Waldrop J. M.; Takeshita T. Y.; Alenaizan A.; Neuhauser D.; King R. A.; Simmonett A. C.; Turney J. M.; Schaefer H. F.; Evangelista F. A.; DePrince A. E. I.; Crawford T. D.; Patkowski K.; Sherrill C. D. Psi4NumPy: An Interactive Quantum Chemistry Programming Environment for Reference Implementations and Rapid Development. J. Chem. Theory Comput. 2018, 14, 3504–3511. 10.1021/acs.jctc.8b00286. 29771539

[ref27] Li R.; Sun Q.; Zhang X.; Chan G. K.-L. Introducing GPU-acceleration into the Python-based Simulations of Chemistry Framework. arXiv 2024, 2407.09700 https://arxiv.org/abs/2407.09700 10.48550/arXiv.2407.09700. PMC11808769 39846468

[ref28] Wu X.; Sun Q.; Pu Z.; Zheng T.; Ma W.; Yan W.; Yu X.; Wu Z.; Huo M.; Li X.; Ren W.; Gong S.; Zhang Y.; Gao W. Enhancing GPU-acceleration in the Python-based Simulations of Chemistry Framework. arXiv 2024, 2404.09452 10.48550/arXiv.2404.09452.

[ref29] Liu W.; Wang F.; Li L. The Beijing density functional (BDF) program package: Methodologies and applications. J. Theor. Comput. Chem. 2003, 02, 257–272. 10.1142/s0219633603000471.

[ref30] Repisky, Komorovsky S.; Malkin V. G.; Malkina O. L.; Kaupp M.; Ruud K., Bast R.; Di Remigio R., Ekstrom U.; Kadek M.; Knecht S.; Konecny L.; Malkin E.; Malkin Ondik I.. ReSpect 5.1.0 Relativistic Spectroscopy DFT Program of Authors M, 2019. (http://www.respectprogram.org). 10.1063/5.0005094 32414255

[ref31] Shiozaki T. BAGEL: Brilliantly Advanced General Electronic-structure Library. Wiley Interdiscip. Rev.: Comput. Mol. Sci. 2018, 8, e1331 10.1002/wcms.1331.

[ref32] Williams-Young D. B.; Petrone A.; Sun S.; Stetina T. F.; Lestrange P.; Hoyer C. E.; Nascimento D. R.; Koulias L.; Wildman A.; Kasper J.; Goings J. J.; Ding F.; DePrince A. E. III; Valeev E. F.; Li X. The Chronus Quantum software package. Wiley Interdiscip. Rev.:Comput. Mol. Sci. 2020, 10, e1436 10.1002/wcms.1436.

[ref33] Pototschnig J. V.; Papadopoulos A.; Lyakh D. I.; Repisky M.; Halbert L.; Severo Pereira Gomes A.; Jensen H. J. A.; Visscher L. Implementation of Relativistic Coupled Cluster Theory for Massively Parallel GPU-Accelerated Computing Architectures. J. Chem. Theory Comput. 2021, 17, 5509–5529. 10.1021/acs.jctc.1c00260. 34370471 PMC8444343

[ref34] Yuan X.; Halbert L.; Pototschnig J. V.; Papadopoulos A.; Coriani S.; Visscher L.; Pereira Gomes A. S. Formulation and Implementation of Frequency-Dependent Linear Response Properties with Relativistic Coupled Cluster Theory for GPU-Accelerated Computer Architectures. J. Chem. Theory Comput. 2024, 20, 677–694. 10.1021/acs.jctc.3c00812. 38193434

[ref35] Lyakh D. I. Domain-specific virtual processors as a portable programming and execution model for parallel computational workloads on modern heterogeneous high-performance computing architectures. Int. J. Quantum Chem. 2019, 119, e25926 10.1002/qua.25926.

[ref36] Kovtun M.; Lambros E.; Liu A.; Tang D.; Williams-Young D. B.; Li X. Accelerating Relativistic Exact-Two-Component Density Functional Theory Calculations with Graphical Processing Units. J. Chem. Theory Comput. 2024, 20, 7694–7699. 10.1021/acs.jctc.4c00843. 39226542

[ref37] Belpassi L.; Storchi L.; Quiney H. M.; Tarantelli F. Recent Advances and Perspectives in Four-Component Dirac-Kohn-Sham Calculations. Phys. Chem. Chem. Phys. 2011, 13, 12368–12394. 10.1039/c1cp20569b. 21670843

[ref38] Quiney H.; Skaane H.; Grant I. Relativistic calculation of electromagnetic interactions in molecules. J. Phys. B:At., Mol. Opt. Phys. 1997, 30, L829 10.1088/0953-4075/30/23/001.

[ref39] Grant I.; Quiney H. Rayleigh-Ritz approximation of the Dirac operator in atomic and molecular physics. Phys. Rev. A 2000, 62, 022508 10.1103/PhysRevA.62.022508.

[ref40] McMurchie L. E.; Davidson E. R. One- and two-electron integrals over cartesian gaussian functions. J. Comput. Phys. 1978, 26, 218–231. 10.1016/0021-9991(78)90092-X.

[ref41] Grant I. P. Relativistic Quantum Theory of Atoms and Molecules: Theory and Computation; Springer Science & Business Media, 2007; Vol. 40.

[ref42] Belpassi L.; Tarantelli F.; Sgamellotti A.; Quiney H. M. Poisson-transformed density fitting in relativistic four-component Dirac-Kohn-Sham theory. J. Chem. Phys. 2008, 128 (12), 124108 10.1063/1.2868770. 18376909

[ref43] Belpassi L.; Tarantelli F.; Sgamellotti A.; Quiney H. M. Electron Density Fitting for the Coulomb Problem in Relativistic Density-Functional Theory. J. Chem. Phys. 2006, 124 (12), 124104 10.1063/1.2179420. 16599659

[ref44] Belpassi L.; Tarantelli F.; Sgamellotti A.; Quiney H. M. All-Electron Four-Component Dirac-Kohn-Sham Procedure for Large Molecules and Clusters Containing Heavy Elements. Phys. Rev. B 2008, 77, 233403 10.1103/PhysRevB.77.233403.

[ref45] Köster A. M.; Reveles J. U.; del Campo J. M. Calculation of exchange-correlation potentials with auxiliary function densities. J. Chem. Phys. 2004, 121, 3417–3424. 10.1063/1.1771638. 15303904

[ref46] Storchi L.; Belpassi L.; Tarantelli F.; Sgamellotti A.; Quiney H. M. An Efficient Parallel All-Electron Four-Component Dirac- Kohn- Sham Program Using a Distributed Matrix Approach. J. Chem. Theory Comput. 2010, 6, 384–394. 10.1021/ct900539m. 26617297

[ref47] Storchi L.; Rampino S.; Belpassi L.; Tarantelli F.; Quiney H. M. Efficient parallel all-electron four-component Dirac–Kohn–Sham program using a distributed matrix approach II. J. Chem. Theory Comput. 2013, 9, 5356–5364. 10.1021/ct400752s. 26592273

[ref48] Rampino S.; Belpassi L.; Tarantelli F.; Storchi L. Full parallel implementation of an all-electron four-component dirac–kohn–sham program. J. Chem. Theory Comput. 2014, 10, 3766–3776. 10.1021/ct500498m. 26588521

[ref49] Belpassi L.; De Santis M.; Quiney H. M.; Tarantelli F.; Storchi L. BERTHA: Implementation of a four-component Dirac–Kohn–Sham relativistic framework. J. Chem. Phys. 2020, 152, 164118 10.1063/5.0002831. 32357778

[ref50] Storchi L.; De Santis M.; Belpassi L. Bertha and pybertha: State of the art for full four-component Dirac-Kohn-Sham calculations. Parallel Computing: Technology Trends; IOS Press, 2020, pp 354–363. 10.3233/APC200060.

[ref51] De Santis M.; Storchi L.; Belpassi L.; Quiney H. M.; Tarantelli F. PyBERTHART: A relativistic real-time four-component TDDFT implementation using prototyping techniques based on Python. J. Chem. Theory Comput. 2020, 16, 2410–2429. 10.1021/acs.jctc.0c00053. 32101419

[ref52] Storchi L.; De Santis M.; Belpassi L. PyBertha project, 2024. https://github.com/BERTHA-4c-DKS/pybertha/(accessed December, 2024 written by: L. Storchi).

[ref53] De Santis M.; Sorbelli D.; Vallet V.; Gomes A. S. P.; Storchi L.; Belpassi L. Frozen-Density Embedding for Including Environmental Effects in the Dirac-Kohn–Sham Theory: An Implementation Based on Density Fitting and Prototyping Techniques. J. Chem. Theory Comput. 2022, 18, 5992–6009. 10.1021/acs.jctc.2c00499. 36172757 PMC9558305

[ref54] GPU Hackathon at CINECA. https://www.openhackathons.org/s/, (accessed: December, 2024).

[ref55] Saunders V. Methods in Computational Molecular Physics; Springer, 1983; pp 1–36.

[ref56] Grant I. P.; Quiney H. M. Application of relativistic theories and quantum electrodynamics to chemical problems. Int. J. Quantum Chem. 2000, 80, 283–297. 10.1002/1097-461X(2000)80:3<283::AID-QUA2>3.0.CO;2-L.

[ref57] Visscher L. Approximate molecular relativistic Dirac-Coulomb calculations using a simple Coulombic correction. Theor. Chem. Acc. 1997, 98, 68–70. 10.1007/s002140050280.

[ref58] Ahmadi G. R.; Almlöf J. The Coulomb operator in a Gaussian product basis. Chem. Phys. Lett. 1995, 246, 364–370. 10.1016/0009-2614(95)01127-4.

[ref59] Challacombe M.; Schwegler E.; Almlöf J. Fast assembly of the Coulomb matrix: A quantum chemical tree code. J. Chem. Phys. 1996, 104, 4685–4698. 10.1063/1.471163.

[ref60] Quiney H. M.; Belanzoni P. Relativistic density functional theory using Gaussian basis sets. J. Chem. Phys. 2002, 117, 5550–5563. 10.1063/1.1502245.

[ref61] De Santis M.; Belpassi L.; Jacob C. R.; Severo Pereira Gomes A.; Tarantelli F.; Visscher L.; Storchi L. Environmental effects with frozen-density embedding in real-time time-dependent density functional theory using localized basis functions. J. Chem. Theory Comput. 2020, 16, 5695–5711. 10.1021/acs.jctc.0c00603. 32786918 PMC8009524

[ref62] Chandra R.; Dagum L.; Kohr D.; Menon R.; Maydan D.; McDonald J. Parallel Programming in OpenMP; Morgan kaufmann, 2001.

[ref63] Repisky M.; Konecny L.; Kadek M.; Komorovsky S.; Malkin O. L.; Malkin V. G.; Ruud K. Excitation energies from real-time propagation of the four-component Dirac–Kohn–Sham equation. J. Chem. Theory Comput. 2015, 11, 980–991. 10.1021/ct501078d. 26579752

[ref64] Konecny L.; Kadek M.; Komorovsky S.; Ruud K.; Repisky M. Resolution-of-identity accelerated relativistic two- and four-component electron dynamics approach to chiroptical spectroscopies. J. Chem. Phys. 2018, 149, 204104 10.1063/1.5051032. 30501232

[ref65] Harris C. R.; Millman K. J.; van der Walt S. J.; Gommers R.; Virtanen P.; Cournapeau D.; Wieser E.; Taylor J.; Berg S.; Smith N. J.; Kern R.; Picus M.; Hoyer S.; van Kerkwijk M. H.; Brett M.; Haldane A.; del Río J. F.; Wiebe M.; Peterson P.; Gérard-Marchant P.; Sheppard K.; Reddy T.; Weckesser W.; Abbasi H.; Gohlke C.; Oliphant T. E. Array programming with NumPy. Nature 2020, 585, 357–362. 10.1038/s41586-020-2649-2. 32939066 PMC7759461

[ref66] Wienke S.; Springer P.; Terboven C.; an Mey D. OpenACC—first experiences with real-world applications. Euro-Par 2012 Parallel Processing: 18th International Conference, Euro-Par 2012, Rhodes Island, Greece, August 27–31, 2012. Proceedings 18, 2012; pp 859–870.

[ref67] Marek A.; Blum V.; Johanni R.; Havu V.; Lang B.; Auckenthaler T.; Heinecke A.; Bungartz H.-J.; Lederer H. The ELPA library: scalable parallel eigenvalue solutions for electronic structure theory and computational science. J. Phys.: Condens. Matter 2014, 26, 213201 10.1088/0953-8984/26/21/213201. 24786764

[ref68] Yu V. W.-z.; Moussa J.; Ks P.; Marek A.; Messmer P.; Yoon M.; Lederer H.; Blum V. GPU-acceleration of the ELPA2 distributed eigensolver for dense symmetric and hermitian eigenproblems. Comput. Phys. Commun. 2021, 262, 107808 10.1016/j.cpc.2020.107808.

[ref69] Tal A.; Marsman M.; Kresse G.; Anders A.; Rodriguez S.; Kim K.; Kalinkin A.; Romanenko A.; Noack M.; Atkinson P. others Solving millions of eigenvectors in large-scale quantum-many-body-theory computations. In ISC High Performance 2024 Research Paper Proceedings (39th International Conference), 2024, pp 1–11.

[ref70] Wolfe M. How compilers and tools differ for embedded systems. In International Conference on Compilers, Architecture and Synthesis for Embedded Systems: Proceedings of the 2005 international conference on Compilers, architectures and synthesis for embedded systems, 2005, p 1.

[ref71] Turisini M.; Amati G.; Cestari M. LEONARDO: A Pan-European Pre-Exascale Supercomputer for HPC and AI Applications. arXiv 2023, 2307.16885 10.48550/arXiv.2307.16885.

[ref72] Dyall K. G. Dyall Dz, Tz, and Qz Basis Sets for Relativistic Electronic Structure Calculations, 2023. 10.5281/zenodo.7574629.

