# Supplementary material for: Acceleration of the Relativistic Dirac–Kohn–Sham Method with GPU: A Pre-Exascale Implementation of BERTHA and PyBERTHA
Source: J Chem Theory Comput. 2025 Mar 21;21(7):3460–75. doi: 10.1021/acs.jctc.4c01759 (PMC11983715; doi:10.1021/acs.jctc.4c01759)
Supplement: Supplementary file 1 — ct4c01759_si_001.pdf [file ct4c01759_si_001.pdf]

Supplementary Information:Acceleration of the relativistic Dirac-Kohn-Sham method with GPU: a pre-exascale implementation of BERTHA and PyBERTHA

Loriano Storchi,[*,†,‡] Laura Bellentani,[¶] Jeff Hammond,[§] Sergio Orlandini,[¶] Nicoló Antonini,[†,‖] and Leonardo Belpassi[*,‡]

†*Dipartimento di Farmacia, Università G. d'Annunzio Chieti-Pescara, via dei Vestini, 66100 Chieti, Italy*

‡ *CNR Institute of Chemical Science and Technologies "Giulio Natta" (CNR-SCITEC), via Elce di Sotto 8, 06123 Perugia, Italy*

¶*CINECA, Via dei Tizi 6/b, 00185 Roma, RM, Italy*

§*NVIDIA Helsinki Oy, 00180 Helsinki, Finland*

‖*Dipartimento di Chimica, Università degli Studi di Perugia, via Elece di Sotto 8, 06123 Perugia, Italy*

E-mail: loriano@storchi.org; leonardo.belpassi@cnr.it

February 24, 2025

1 Molecular systems: basis set, fitting set and geometry

For Au the large component of the basis set was generated by uncontracting double-ζ quality Dyall's basis sets [1–4] augmented with the related polarization and correlating functions, while the corresponding small component basis was generated using the restricted kinetic balance relation. The final basis set scheme is : Au $(24s19p12d9f)$. Large component basis functions for O were derived by decontracting the related def2-svpd basis set available at the "Basis Set Exchange" site .[5] The corresponding small component basis was generated using the restricted kinetic balance relation.[6] For gold the B16 optimized auxiliary basis sets been used.[7] For the O element, accurate auxiliary basis set were generated using a simple procedure starting from available DeMon Coulomb fitting basis set. The final sets are referred as Demon A2.[8] For the sake of clarity we give the dimension of the O-atom auxiliary basis sets, which is (9s,4p,1d,1f,1g). Finally a BLYP functional were used.[9,10] An energy convergence criterion of 10^{-7} Hartree on the total energy was adopted.

Table S1: H_2O molecule geometry

Atom	X (a.u.)	Y (a.u.)	Z (a.u.)
H	10.00	6.38	0.00
H	10.31	9.29	0.00
O	11.29	7.71	0.00

Table S2: Au_2 gold cluster geometry

Atom	X (a.u.)	Y (a.u.)	Z (a.u.)
Au	0.00	0.00	0.00
Au	0.00	0.00	4.82

Table S3: Au_4 gold cluster geometry

Atom	X (a.u.)	Y (a.u.)	Z (a.u.)
Au	0.00	0.00	0.00
Au	0.00	0.00	4.82
Au	6.00	0.00	0.00
Au	6.00	0.00	4.82

Table S4: Au_8 gold cluster geometry

Atom	X (a.u.)	Y (a.u.)	Z (a.u.)
Au	0.00	0.00	0.00
Au	0.00	0.00	4.82
Au	6.00	0.00	0.00
Au	6.00	0.00	4.82
Au	0.00	4.82	0.00
Au	0.00	4.82	4.82
Au	6.00	4.82	0.00
Au	6.00	4.82	4.82

Table S5: Au_{16} gold cluster geometry

Atom	X (a.u.)	Y (a.u.)	Z (a.u.)
Au	0.00	0.00	0.00
Au	0.00	0.00	4.82
Au	6.00	0.00	0.00
Au	6.00	0.00	4.82
Au	0.00	4.82	0.00
Au	0.00	4.82	4.82
Au	6.00	4.82	0.00
Au	6.00	4.82	4.82
Au	0.00	9.64	0.00
Au	0.00	9.64	4.82
Au	6.00	9.64	0.00
Au	6.00	9.64	4.82
Au	0.00	14.46	0.00
Au	0.00	14.46	4.82
Au	6.00	14.46	0.00
Au	6.00	14.46	4.82

References

(1) Dyall, K. G. Relativistic double-zeta, triple-zeta, and quadruple-zeta basis sets for the 5d elements Hf–Hg. *Theoretical Chemistry Accounts* **2004**, *112*, 403–409.

(2) Dyall, K. G.; Gomes, A. S. Revised relativistic basis sets for the 5d elements Hf–Hg. *Theoretical Chemistry Accounts* **2010**, *125*, 97.

(3) Dyall, K. G. Relativistic and nonrelativistic finite nucleus optimized triple-zeta basis sets for the 4p, 5p and 6p elements. *Theoretical Chemistry Accounts* **2002**, *108*, 335–340.

(4) Dyall, K. G. Relativistic quadruple-zeta and revised triple-zeta and double-zeta basis sets for the 4p, 5p, and 6p elements. *Theoretical Chemistry Accounts* **2006**, *115*, 441–447.

(5) Schuchardt, K. L.; Didier, B. T.; Elsethagen, T.; Sun, L.; Gurumoorthi, V.; Chase, J.; Li, J.; Windus, T. L. Basis set exchange: a community database for computational sciences. *Journal of chemical information and modeling* **2007**, *47*, 1045–1052.

(6) Grant, I.; Quiney, H. Rayleigh-Ritz approximation of the Dirac operator in atomic and molecular physics. *Phys. Rev. A* **2000**, *62*, 022508.

(7) Belpassi, L.; Tarantelli, F.; Sgamellotti, A.; Quiney, H. M. Electron Density Fitting for the Coulomb Problem in Relativistic Density-Functional Theory. *J. Chem. Phys.* **2006**, *124*, 124104 (8).

(8) De Santis, M.; Rampino, S.; Storchi, L.; Belpassi, L.; Tarantelli, F. The Chemical Bond and s–d Hybridization in Coinage Metal (I) Cyanides. *Inorg. Chem.* **2019**, *58*, 11716–11729.

(9) Becke, A. D. Density-functional exchange-energy approximation with correct asymptotic behavior. *Physical review A* **1988**, *38*, 3098.

(10) Lee, C.; Yang, W.; Parr, R. Phys. l rev. b 37, 785 (1988);(b) ad becke. *Phys. Rev. A* **1988**, *38*, 3098.
